# Acid-responsive nanoparticles with microneedles for anti‑inflammatory sonodynamic therapy of gouty arthritis

**DOI:** 10.1080/10717544.2026.2743337

**Published:** 2026-10-13

**Authors:** Jiaqi Weng, Lili Feng, Binghui Xie, Linxiao Liu, Shulin Shen, Xuechao Chen, Liting Wang, Yueyue Xie, Jianhua Yao, Laurent David, Qinying Yan

**Affiliations:** a College of Mechanical Engineering, Zhejiang University of Technology, Hangzhou, PR China; b Institute of Laser Advanced Manufacturing, Zhejiang University of Technology, Hangzhou, PR China; c College of Pharmaceutical Sciences, Zhejiang University of Technology, Hangzhou, PR China; d Department of Ophthalmology, Shanghai Ninth People’s Hospital, Shanghai Jiao Tong University School, Shanghai, PR China; e Ingénierie des Matériaux Polymères, Université Lyon 1, INSA Lyon, Université Jean Monnet, CNRS, UMR 5223, Villeurbanne, France

**Keywords:** Gouty arthritis, sonodynamic therapy, curcumin, acid-sensitive drug delivery, microneedle drug delivery system

## Abstract

The pathological microenvironment of acute gouty arthritis (AGA) features local tissue acidification and the release of a storm of pro-inflammatory cytokines. To address this dual challenge, we engineered an acid-responsive curcumin-loaded nanoparticles microneedle system (Cur-PNPs@MN) that delivers curcumin with pH-triggered release and anti-inflammatory activity. This integrated platform combines a hyaluronic acid (HA)-based dissolving microneedle for localized transdermal delivery of acid-degradable PCADK (poly (cyclohexane-1,4-diyl acetone dimethylene ketal)) curcumin nanoparticles (Cur-PNPs), which are designed for pH-triggered curcumin release at the pathological site. Together, they deliver curcumin, a dual-function agent acting both as an anti-inflammatory compound and a nanoenhanced sonosensitizer. *In vitro*, the system exhibited acid-responsive drug release and also promoted macrophage uptake, achieving significant curcumin accumulation within 1 hour and saturation by 4 hours. Moreover, it could generate potent ultrasound-induced reactive oxygen species (ROS). The ROS generation capability of Cur-PNPs was 1.47-fold higher than that of Cur‑aggregates, resulting in anapoptosis rate of 29.9% in M1 macrophages. *In vivo,* the chemo-sonodynamic therapy (Cur-PNPs@MN+Ultrasound (US)) in a rat AGA model significantly alleviated joint swelling and inflammatory infiltration, while drastically suppressing pro-inflammatory cytokines, with IL-1β decreasing from 170.3 to 67.3 pg/mL, TNF-*α* from 20.8 to 5.9 pg/mL, and IL-6 from 140.9 to 48.2 pg/mL, and achieved therapeutic efficacy comparable to conventional drugs. This work demonstrates a synergistic chemo‑sonodynamic strategy by coupling pH-triggered curcumin release with ultrasound-enhanced ROS generation. This acid-responsive therapeutic strategy enhances anti-inflammatory efficacy and reduction of M1-associated inflammatory responses, offering an effective approach for AGA therapy.

## Graphical abstract

**Figure:**
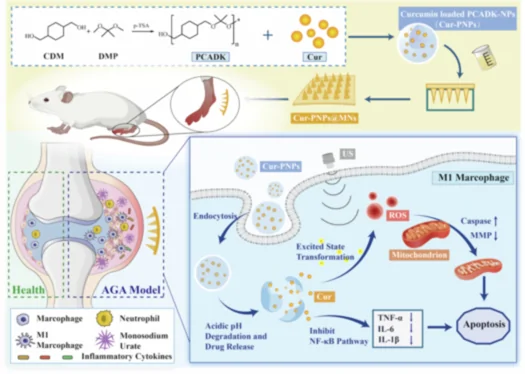

## 1. Introduction

Gouty arthritis is an acute inflammation caused by the accumulation of monosodium urate (MSU) crystals in joints and surrounding tissues. During the pathological progression of acute gouty arthritis (AGA), the local microenvironment demonstrates marked acidification and accumulation of high concentrations of inflammatory cytokines, such as interleukin-1β (IL-1β) and tumor necrosis factor-*α* (TNF-*α*) (Shin et al. 2019; Huang and Chiang 2021). The crucial mechanism is the activation of the NLRP3 (NOD−, LRR− and pyrin domain-containing protein 3) inflammasome by MSU crystals *via* the NF-κB (NF-κB (Nuclear Factor Κ-light-chain-enhancer of activated B cells) signaling pathway, which triggers a severe immune response in the joints (Liu et al. 2023; Tao et al. 2023; Xiao et al. 2024). In this process, macrophage polarization toward the pro-inflammatory M1 phenotype promotes further production and recruitment of inflammatory factors, causing the infiltration and activation of neutrophils and triggering a sustained and excessive inflammatory response, which is a key to AGA (Chen et al. 2024; Tan et al. 2024). According to these findings, M1-polarized macrophage interference is a critical therapeutic target for AGA.

Colchicine, nonsteroidal anti-inflammatory medications (NSAIDs), or glucocorticoids are commonly used in conventional therapy techniques for AGA (Lee et al. 2019). They primarily exert their therapeutic effects by suppressing the inflammatory response involved in the disease process. Colchicine, for example, modulates the inflammatory response by inhibiting M1 macrophage polarization, neutrophil recruitment, and inflammasome activation (Fine et al. 2020; Chen et al. 2024). However, it is well established that oral administration of NSAIDs causes significant gastrointestinal irritation and is frequently associated with urticaria, angioedema, allergic reactions, and severe systemic organs damage (Aun et al. 2014; Bindu et al. 2020; Lee 2024). Furthermore, the anti-inflammatory action of NSAIDs by cyclooxygenase (COX) may impair the natural anti-inflammatory function of the COX-PGE2 pathway, potentially aggravating inflammation with prolonged use (Cha and DuBois 2007; Tanaka et al. 2009; Sokolowska et al. 2015). Thus, the clinical treatment of gout requires alternatives to NSAIDs with high targeting ability, minimal side effects, and low long-term toxicity.

Sonodynamic therapy (SDT) employs sonosensitizers to generate cytotoxic reactive oxygen species (ROS) under ultrasound irradiation (Liang et al. 2020, 2023). These ROS directly induce inflammatory cell damage or apoptosis, offering a distinct mechanism that bypasses the COX-PGE2 pathway inhibition associated with NSAIDs. This mechanism offers several key advantages including spatiotemporally controllable action, target-selective cytotoxicity, and minimally invasive safety. Thus, SDT has emerged as a promising alternative in oncology, atherosclerosis, and AGA (Cao et al. 2022; Dong et al. 2023; Zhao et al. 2024; Wu et al. 2024a). The efficacy of SDT varies significantly across different systems, largely attributable to the highly heterogeneous immunological microenvironment within the joint cavity (Zhou et al. 2024; Wu et al. 2024a). The efficacy of SDT is primarily determined by the performance of the sonosensitizer, particularly its bioactivity and spatiotemporal distribution (Yan et al. 2020). Notably, macrophages enhance cellular uptake efficiency through receptor-mediated endocytosis, significantly improving sonosensitizer bioactivity. This makes them one of the most suitable biological targets for SDT (Alam et al. 2017; Xin Li et al. 2023; Soni et al. 2024). Moreover, their active transport capability optimizes spatiotemporal delivery of sonosensitizer to disease sites, further enhancing therapeutic efficacy.

Curcumin, a natural polyphenolic compound derived from the rhizome of Curcuma longa, combines excellent biocompatibility and various bioactivities, including anti-inflammatory, antioxidant, and antitumor properties (Hasanzadeh et al. 2020; Peng et al. 2021; Zhang et al. 2022b). It suppresses the NF-κB pathway and downstream inflammatory cytokines, thereby inhibiting M1 macrophage polarization and NLRP3 inflammasome-driven inflammatory cascades (Mohammadi et al. 2019; Hasanzadeh et al. 2020; Zhang et al. 2022a; Dai et al. 2023). However, this anti-inflammatory effect is predominantly cytostatic; it suppresses the inflammatory activity of macrophages without eliminating the pathogenic cells themselves. Notably, curcumin also functions as a natural sonosensitizer, generating ROS under specific ultrasound irradiation, representing an additional, distinct mechanism beyond its anti-inflammatory activity (Xu et al. 2020). Curcumin can selectively disrupt mitochondrial integrity in internalized M1 macrophages, promoting their apoptosis and facilitating homeostatic microenvironment restoration (Jiang et al. 2020). This dual mechanism functional (suppression combined with physical elimination) offers a synergistic advantage over either monotherapy alone. The chemotherapy component provides rapid anti-inflammatory relief, while the SDT component ensures sustained resolution by removing the cellular source of inflammation. Furthermore, the acid-responsive release of curcumin from PCADK nanoparticles ensures that both therapeutic actions are localized to the acidic inflammatory microenvironment, maximizing efficacy while minimizing systemic toxicity.

This chemo-sonodynamic dual-action offers potent M1 clearance and synergistic therapy for AGA. However, curcumin's clinical translation is hindered by poor solubility, instability, and nonspecific distribution (Prasad et al. 2014; Liu et al. 2024). To overcome these challenges, nanocarriers tailored to pathological features have been developed, including exosome-coated calcium carbonate nanoparticles for colon cancer (Li et al. 2022), acid-sensitive mesoporous silica nanoparticles for hepatocellular carcinoma (Zhang et al. 2024), and cell membrane-coated PLGA (poly- D,L-lactide-co-glycolide) nanoparticles for breast cancer (Liao et al. 2025). These studies collectively demonstrate that tailoring nanocarriers to disease-specific microenvironments can substantially improve curcumin's therapeutic performance. Building on this concept, curcumin-based nanocarriers have also been developed for various inflammatory conditions. For instance, a theranostic curcumin-loaded nanoemulsion was shown to suppress macrophage polarization toward the M1-like phenotype, decreasing the release of pro-inflammatory cytokines while increasing IL-10 levels in a rat model of acute inflammation (Vichare et al. 2025). Chondroitin sulfate-modified tragacanth gum–gelatin composite nanocapsules loaded with curcumin nanocrystals demonstrated CD44-mediated joint targeting and significantly alleviated joint swelling in arthritis models (Sun et al. 2024). Hyaluronic acid-based dissolving microneedles have also been employed to deliver curcumin nanoformulations for transdermal treatment of inflammatory skin disorders (Cheng et al. 2020). These examples collectively highlight the feasibility of utilizing curcumin nanocarriers for inflammatory diseases.

The unique pathological milieu of AGA, characterized by tissue acidification and cytokine storms, demand a acid-targeting strategy integrating pH-triggered drug release and rapid anti-inflammatory action (Huang and Chiang 2021; Tao et al. 2023). PCADK is an acid-sensitive polymer containing ketal bonds in its backbone, which remain stable at physiological pH but undergo rapid hydrolysis under acidic conditions (pH 4.5–6.5). This property makes PCADK particularly suitable for the AGA microenvironment, where local tissue acidification can serve as an endogenous trigger for drug release. Moreover, PCADK exhibits tunable hydrolysis kinetics (48-hour half-life at pH 4.5) and is well suited to the acidic endosomal environment of disease-driving M1 macrophages during AGA progression (Lee et al. 2007; Zhao et al. 2021). This property enhances drug accumulation at target sites while minimizing off-target toxicity.

In this study, hyaluronic acid-based (Hyaluronic acid has excellent biocompatibility, intrinsic CD44-mediated anti-inflammatory activity, rapid dissolution upon skin insertion, and sufficient mechanical strength for effective skin penetration) microneedles (HA-MN) were employed to create transdermal microchannels that bypass systemic circulation, enabling more rapid attainment of therapeutic drug concentrations at local disease sites for direct intervention in the inflammatory microenvironment of acute gouty arthritis (Du et al. 2021; Diao et al. 2025). Recognizing the central role of M1 macrophages in AGA pathogenesis, we developed an integrated chemo-sonodynamic therapy using the Cur-PNPs@MN system. This platform combines curcumin-loaded PCADK nanoparticles (Cur-PNPs) with HA-MN technology to achieve improved spatiotemporal control over delivery. Our data show that the system enables efficient cell targeting, enhances ROS generation, and selective M1 cytoreduction via apoptosis. In an AGA model, treatment reduced both inflammatory and acidic microenvironmental components with an acceptable safety profile. Notably, although the affected joints are typically painful, the microneedle length (<500 μm) limits penetration to the stratum corneum, and the low-intensity pulsed sonication is well tolerated without sedation or general anesthesia. It is important to emphasize that this approach targets acute inflammatory flares rather than the underlying urate deposition; long-term disease control still requires urate-lowering therapy. Thus, AGA is used here as a model inflammatory disease to establish proof-of-concept. This microenvironment-adaptive strategy may also be applicable to other inflammation-driven conditions involving M1 macrophage polarization, such as rheumatoid arthritis (Yang et al. 2025), psoriatic arthritis, and cutaneous lupus (Cutolo et al. 2025).

## 2. Materials and methods

### 2.1. Materials

Curcumin (≥98%, Cat. No. 58426B, damas-beta, USA) was purchased from damas-beta. PCADK monomers, including 1,4-cyclohexanedimethanol (99%, Cat. No. C761311), and 2,2-dimethoxypropane (99%, Cat. No. D806894), *p*-toluenesulfonic acid (99%, Cat. No. T823839) were purchased from Macklin Biochemical, Shanghai, China. Other analytical-grade chemicals, such as dichloromethane (AR ≥ 99.5%, Cat. No. 40013564), acetone (HPLC ≥ 99.7%, Cat. No. 40064460), methanol (HPLC ≥ 99.8%, Cat. No. 40064260), acetonitrile (HPLC ≥ 99.8%, Cat. No. 40064160), polyvinyl alcohol (Cat. No. XW01900289504), trehalose (99%, Cat. No. 63012664), sodium carbonate (AR ≥ 99.8%, Cat. No. 10019260), sodium bicarbonate (AR ≥ 99.5%, Cat. No. 10018960), gelatin (Cat. No. 10019260), hyaluronic acid (95%, Cat. No. XW01906732701), and phosphate-buffered saline (PBS) (Cat. No. 72013561), were supplied by Sinopharm Chemical Reagent, Shanghai, China. Fluorescent probes including 1,3-diphenylisobenzofuran (DPBF) (97%, Cat. No. Y079423), 9,10-anthracenediyl-bis(methylene) dimalonic acid (ABDA) (≥90%, Cat. No. Y233641), and ROS assay kits (Cat. No. S0034S) were obtained from Beyotime Biotechnology, Shanghai, China. Annexin V-APC/7-AAD Apoptosis detection kits (E-CK-A218) were purchased from Procell, Wuhan, China. 4',6-diamidino-2-phenylindole (DAPI) staining solution (Cat. No. ID22502) and 4% paraformaldehyde (Cat. No. P1110) were supplied by Solarbio, Beijing, China.

RAW264.7 murine macrophage cells (CSTR:19375.09.3101MOUTCM13) and L929 fibroblast cells (5301MOU-KCB92012YJ) were obtained from the Cell Bank of the Chinese Academy of Sciences. Cells were cultured in high-glucose DMEM (Cat. No. 11995) supplemented with 10% fetal bovine serum (Cat. No. S9030) and 1% penicillin–streptomycin (Cat. No. P1400) were obtained from Solarbio, Beijing, China.

Male Sprague–Dawley rats (200 ± 20 g, 6–8 weeks old) were purchased from the Laboratory Animal Center of Zhejiang Academy of Medical Sciences (Hangzhou, China). Animals were housed under specific pathogen-free (SPF) conditions and were confirmed to be in good health prior to the study.

### 2.2. Synthesis and characterization of PCADK

#### 2.2.1. Synthesis PCADK

Using 1,4-cyclohexanedimethanol and 2,2-dimethoxypropane as comonomers, the acid-sensitive polymer PCADK was synthesized through the ketal exchange reaction, as illustrated in Figure 1. Specifically, 12.5 g of 1,4-cyclohexanedimethanol (CDM) was dissolved in 70 mL of benzene and heated to 105 °C until the volume reduced to approximately 50 mL. Subsequently, 16.5 g of *p*-toluenesulfonic acid (*p*-TSA) was added with magnetic stirring, and the temperature was adjusted to 95 °C. Then, 10.8 mL of 2,2-dimethoxypropane (DMP) was added dropwise to initiate the reaction. During the 3-hours reaction, additional 2,2-dimethoxypropane (2 mL each time, 6 times) and benzene were supplemented hourly to maintain a constant volume. The overall feed molar ratio of DMP to CDM was approximately 2:1. The excess DMP served to drive the ketal exchange equilibrium toward completion, ensuring near‑quantitative conversion of both hydroxyl groups of CDM into cyclic ketal units.

After the reaction, the temperature was raised to 105 °C to evaporate benzene and unreacted DMP. The mixture was then cooled to 60 °C, and the reaction was quenched with 0.45 mL of triethylamine. The product was precipitated in cold *n*-hexane, collected by suction filtration, and vacuum-dried for 24 hours to obtain a pale yellow solid powder. This extended drying under reduced pressure effectively removes residual benzene, as supported by FTIR spectroscopy (see Section 3.1), which shows no aromatic C–H stretching (~3030 cm^−1^) or C═C stretching (~1600 cm^−1^) peaks. The structure and crystallinity of PCADK were further confirmed by ^1^H NMR, FTIR, and XRD (Section 3.1).

**Figure 1.**
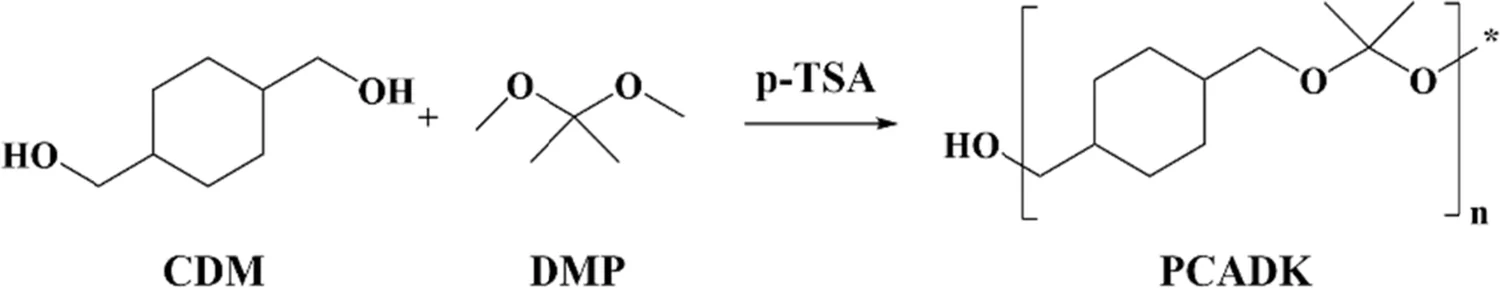
Synthesis route of PCADK.

#### 2.2.2. Characterization of PCADK

**Proton nuclear magnetic resonance (^1^H NMR) spectroscopy:** A 5.0 mg sample of PCADK was accurately weighed into a dedicated NMR tube, and 0.5 mL of deuterated chloroform (CDCl₃) was added as the solvent. After complete dissolution, the sample was analyzed using a nuclear magnetic resonance spectrometer (HD600Hz Bruker AVANCE III, Field:14.09 T). The structure was verified by examining the chemical shifts and peak patterns of the characteristic hydrogen atoms.

**Fourier transform infrared (FTIR) spectroscopy:** A 2.0 mg quantity of PCADK powder was accurately weighed and ground with potassium bromide at a mass ratio of 1:100 (sample:potassium bromide) to obtain a fine powder. The resulting mixture was compressed into a pellet using a tablet press and subsequently analyzed using a Fourier transform infrared spectrometer (Nicolet 6700, Thermo Fisher Scientific, with 32 scans per spectrum) over the range of 400–4000 cm^−1^.

**X-ray Diffraction (XRD):** An approximately 200 mg of PCADK sample was thoroughly ground in a mortar. The powdered sample was loaded onto a zero‑background quartz sample holder (non‑crystalline, to avoid extraneous diffraction peaks) using the flat-plate method and leveled with a glass slide. XRD patterns were recorded on an ARL EQUINOX X‑ray diffractometer (Thermo Fisher Scientific, USA) equipped with a Cu Kα radiation source (*λ* = 1.54056 Å). Data were collected over a 2θ range of 5–50° with a step size of 0.02° and a scan speed of 2°/min.

### 2.3. Preparation and characterization of Cur-PNPs

#### 2.3.1. Preparation of Cur-PNPs

Cur-PNPs were prepared using the emulsion solvent evaporation method. Briefly, PCADK and Cur were weighed (100 mg PCADK and 50 mg Cur) and dissolved in a mixed solvent of dichloromethane and acetone to obtain the organic phase (2 mL dichloromethane and 1 mL acetone). The organic phase was added dropwise to a 10 mL of 1% PVA aqueous phase, and the mixture was sonicated for 5 min in an ice bath to obtain the primary emulsion. Subsequently, the primary emulsion was transferred into 0.5% (*w/v*) PVA solution and stirred magnetically at room temperature for 4 hours to allow complete organic solvent evaporation. The obtained nanoparticle suspension was centrifuged at 5000 rpm (Thermo Scientific X1R centrifuge, fixed‑angle rotor, rotor radius ≈ 8.5 cm) at 4 °C for 10 minutes to remove Cur‑aggregates. The supernatant containing Cur-PNPs was collected and stored at 4 °C in the dark. For lyophilization, 4% (*w/v*) D-trehalose was added as a cryoprotectant, and the dispersion was pre-frozen at −80 °C overnight before freeze-drying to obtain a powdered form.

#### 2.3.2. Characterization of Cur-PNPs

**Particle size and morphology:** the particle size and polydispersity index (PDI) of the freshly prepared Cur-PNPs suspensions (before lyophilization) were determined at 25 °C by dynamic light scattering (DLS) (Zetasizer Nano ZS90, Malvern, UK). Briefly, Cur-PNPs nanosuspensions were diluted to 1 mg/mL with deionized water. A total of 1 mL of each solution sample was pipetted into a particle size cuvette and placed in a Malvern particle size analyzer for detection.

Each batch was analyzed in triplicate. the morphology of nanoparticles was also examined by transmission electron microscopy (TEM) and scanning electron microscopy (SEM). To this end, Cur-PNPs suspension was prepared at a concentration of 1 mg/mL, and then the diluted suspension was drop-cast onto a copper grid. Excess liquid at the edges of the copper grid was blotted away with filter paper; the morphologicy of Cur-PNPs were observed after air-drying.

**Crystallinity analysis:** the crystalline states of the samples (5 mg each of Blank-PNPs, Cur-PNPs and Cur‑aggregates powder) were characterized by X-ray diffraction (XRD) on a Smart Lab diffractometer (Rigaku Corporation, Tokyo, Japan) operating at 40 kV and 40 mA with Cu Kα radiation (*λ* = 1.5406 Å). The samples were loaded onto a low-background silicon sample holder, and diffractograms were recorded over a 2θ range of 5–50° at a scan speed of 2°/min and a step size of 0.02°. The encapsulation of curcumin within the PCADK nanoparticles was confirmed by comparing the XRD patterns.

**Evaluation of encapsulation efficiency (EE) of Cur-PNPs:** 1 mL of the Cur-PNPs suspension was centrifuged at 10,000 rpm for 20 minutes at 4 °C (under these conditions, the applied centrifugal force is sufficient to sediment the ~236 nm Cur-PNPs). It should be noted that this high-speed centrifugation serves a different purpose from the low-speed centrifugation (5000 rpm, 10 minutes) used in the preparation step (Section 2.3.1), which was designed solely to remove large aggregates and unencapsulated curcumin precipitates while leaving the well-dispersed Cur-PNPs in the supernatant. The supernatant was collected, and 100 μL was diluted with 900 μL of acetone (10‑fold dilution, final acetone/water ratio ≈ 90:10). Separately, 100 μL of the original Cur-PNPs suspension was also diluted with 900 μL of acetone to completely disrupt the nanoparticles and release all encapsulated curcumin. The absorbance of both samples was measured at 420 nm using a UV-vis spectrophotometer. The concentrations of curcumin were calculated based on a standard calibration curve of curcumin in acetone. And the EE% was calculated using the formula (1):

(1) $$\mathrm{EE}(\%)=\frac{C_{\mathrm{total}-}C_{\mathrm{supernatant}}}{C_{\mathrm{total}}}\times 100\%$$

where ${\text{C}}_{\text{total}}$ QUOTEand $C_{\mathrm{supernatant}}$ QUOTE are the curcumin concentrations in the original suspension and the supernatant, respectively.

**The drug loading content (DLC) of Cur-PNPs:** the Cur-PNPs suspension was centrifuged at 10,000 rpm for 20 minutes at 4 °C, and the supernatant was collected. The curcumin concentration in the supernatant was determined by UV-vis spectroscopy at 420 nm. The pellet obtained after centrifugation was washed with deionized water, lyophilized, and weighed. The lyophilized nanoparticles were then disrupted with a small amount of dichloromethane, and curcumin was extracted with 1 mL of acetone. The DLC was calculated using the formula (2):

(2) $$\mathrm{DLC}(\%)=\frac{C_{\mathrm{curcumin}}\times V_{\mathrm{dilution}\;\mathrm{factor}}}{M_{\mathrm{lyophilized}}}$$

where ${\text{C}}_{\text{curcumin}}$ is the concentration of curcumin in the extracted solution, $V_{\mathrm{dilution}\;\mathrm{factor}}$ accounts for dilution, and the ${\text{M}}_{\text{lyophilized}}$ is the mass of lyophilized nanoparticle.

**Colloidal stability analysis:** Cur-PNPs were suspended in phosphate-buffered saline (PBS) of pH 7.4 and then incubated at 4 ± 2 °C and 28 ± 2 °C. The sizes and polydispersity indexes of the Cur-PNPs were measured using DLS at predetermined time points to evaluate their stability.

#### 2.3.3. The release of Cur in vitro

The release of Cur from freshly prepared nanoparticle suspensions (before lyophilization) nanoparticles was investigated by incubation of nanoparticles in PBS at pH 7.4, 6.5 and 5.0. Briefly, 2 mL of the freshly prepared nanoparticle suspension was added into dialysis bags (MWCO: 100 kDa, significantly higher than the molecular weight of Cur‑aggregates), which were then immersed in 30 mL of release medium. The samples were incubated at 37 °C in a constant-temperature shaking incubator at 100 rpm for 48 hours. At pre-specified time intervals, 2 mL of supernatant was collected and replaced with an equal volume of fresh PBS. The Cur concentrations were measured by UV-vis spectroscopy (420 nm).

#### 2.3.4. Investigation on the sonodynamic activity of Cur-PNPs in vitro

**General ROS detection:** the ROS-generating abilities of Cur‑aggregates and Cur–PNPs were evaluated using 1,3-Diphenylisobenzofuran (DPBF, Sigma-Aldrich, USA) as a ROS indicator. DPBF reacts predominantly with singlet oxygen (^1^O₂), but can also react with hydroxyl radical (•OH) and superoxide (O₂•^-^) to a lesser extent. Briefly, 10 mL suspensions of Cur-PNPs at concentrations of 5, 10, and 20 μg/mL were prepared. For a comparison, a 10 mL aqueous dispersion of Cur‑aggregates (equivalent to the curcumin mass in 10 μg/mL Cur-PNPs) was also prepared by dissolving curcumin in a minimal volume of acetone and rapidly diluting with deionized water under vigorous stirring (this dispersion is metastable but sufficient for short-term measurement). Then, 50 μL of a 10 mM DPBF solution (in DMSO) was added to each sample. After thorough mixing, the mixtures were irradiated with low-frequency focused ultrasound (1 MHz, 1 W/cm^2^) in the dark. The spatial average-temporal average (SATA) intensity was determined by measuring the total acoustic power of the transducer with a radiation force balance and dividing by its effective radiating area (ERA). The decrease in DPBF absorbance at 427 nm was monitored every minute using UV-vis spectroscopy to determine ROS generation efficiency. A control experiment was performed by adding the same amount of DPBF to 10 mL deionized water. All experiments were conducted in triplicate. Moreover, in order to study the effect of ultrasound power on ROS generation, a 10 μg/mL Cur-PNPs suspension was tested under varying ultrasound intensities (0, 0.5, 1, 1.5, and 2 W/cm^2^), with other conditions kept constant.

**Singlet oxygen (^1^O₂) detection:** the singlet oxygen (^1^O₂)-generating ability was assessed using 9,10-anthracenediyl-bis(methylene)dimalonic acid (ABDA, Aladdin, China) which selectively reacts with ^1^O₂. A 10 mL suspension of 10 μg/mL Cur-PNPs and an equivalent Cur‑aggregates dispersion (matched in curcumin content, calculated based on the drug loading content) were prepared. Then, 100 μL of a 1.5 mg/mL ABDA solution (in DMSO) was added to each sample. After mixing, the solutions were sonicated (1 MHz, 1 W/cm^2^, SATA) in the dark. The decay of ABDA absorbance at 400 nm was measured every minute by UV-Vis spectroscopy to evaluate ^1^O₂ generation. A control (ABDA in deionized water) was included, and all experiments were performed in triplicate.

### 2.4. Preparation and characterization of Cur-PNPs@MN

#### 2.4.1. Preparation of Cur-PNPs@MN

As shown in Figure 2, the soluble microneedle patch loaded with curcumin nanoparticles was fabricated using an inverse molding technique. The process started with the preparation of the microneedle matrix, which was formed by homogeneously mixing lyophilized Cur-PNPs powder with hyaluronic acid (HA) to obtain a 10% (*w/v*) HA solution incorporating Cur-PNPs (each patch contains about 3.0 mg of Cur-PNPs). Subsequently, the drug-loaded matrix was introduced into a polydimethylsiloxane (PDMS) negative microneedle mold (10 × 10 array). The filled mold was subjected to centrifugation at 3500 rpm for 15 minutes, followed by inversion and further centrifugation for an additional 15 minutes to ensure complete infiltration of the microneedle cavities. Excess material was carefully scraped off, and an additional 150 μL of the microneedle matrix was uniformly applied onto the mold surface. A stepwise centrifugation protocol was implemented to optimize microneedle formation: initial centrifugation at 3000 rpm for 5 minutes was followed by sequentially reduced speeds of 2500, 2000, 1500, 1000, 750, and finally 500 rpm, each for 5 minutes. This gradual reduction in centrifugal force produced microneedles with a smooth and flat backing layer. Finally, the microneedles were dried at 40 °C and 30% relative humidity under a controlled environment to obtain the finished Cur-PNPs@MN patch.

**Figure 2.**
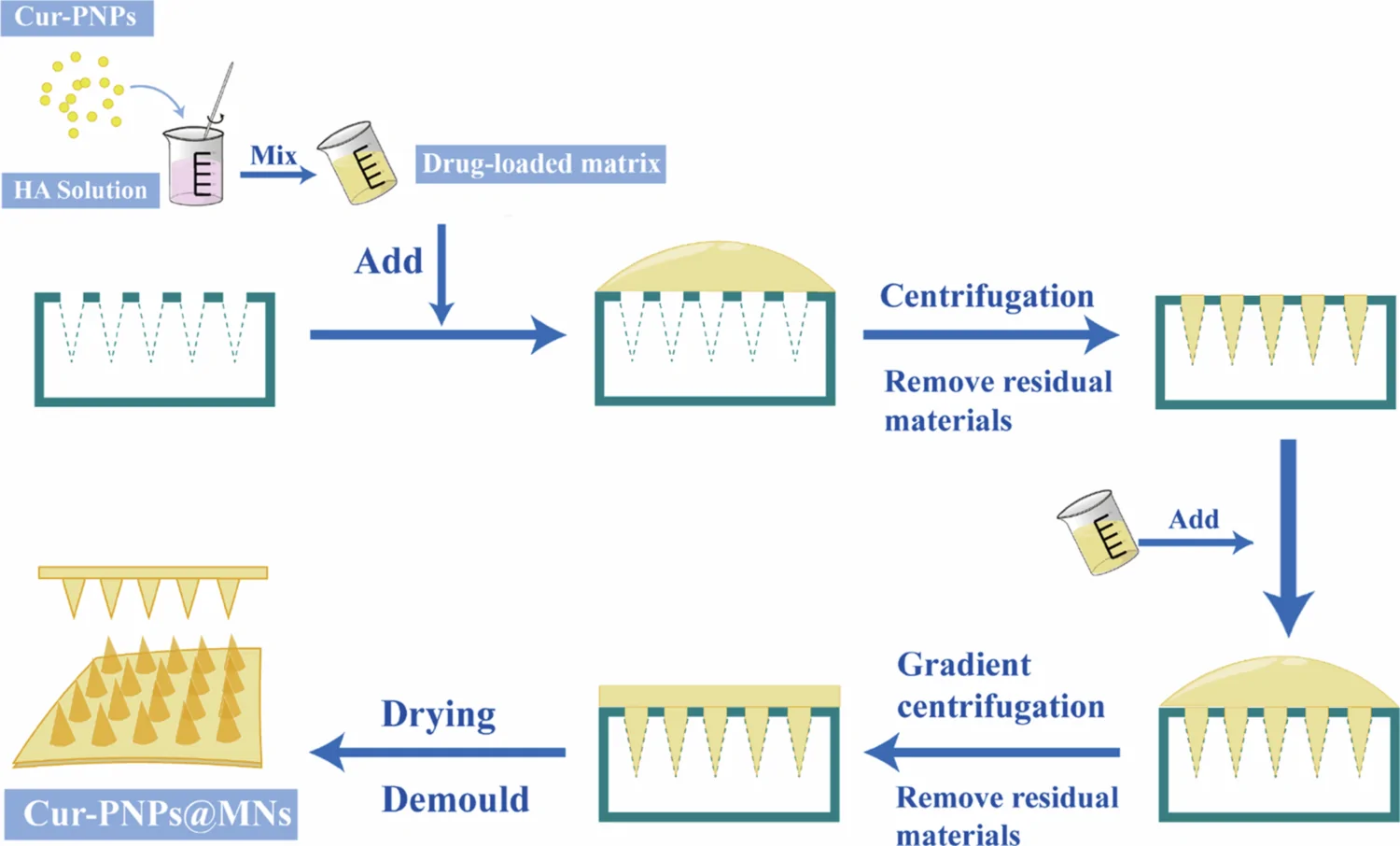
Flow chart for preparation of microneedles.

#### 2.4.2. Characterization of Cur-PNPs@MN

**Morphological characterization of microneedles:** the morphology of the microneedles was initially examined using digital photography and optical microscopy, followed by detailed structural characterization using scanning electron microscopy (SEM). Randomly selected microneedles were mounted on an aluminum stub using conductive carbon tape and sputter-coated with a thin layer of gold. The surface morphology was then observed under high vacuum conditions.

**Mechanical property evaluation:** the mechanical properties were evaluated by measuring the critical buckling force using a texture analyzer in compression mode. To assess skin compression behavior, forces ranging from 0.5 *N* to 2 *N* were applied to 2 × 2 microneedle arrays while monitoring morphological changes and height reduction percentages under an optical microscope.

***In vivo* performance assessment:** for *in vivo* evaluation, the microneedles were inserted into treated mouse skin for 60 seconds, after which the application site was stained with 0.4% trypan blue solution for 3–5 minutes to determine puncture efficiency. Drug penetration was evaluated in excised rat skin by utilizing curcumin's fluorescence evaluated with confocal laser scanning microscopy.

**Dissolution profile analysis:** the dissolution profile was characterized by observing the swelling and dissolution dynamics of 1 × 10 microneedle arrays in PBS (pH 6.5) under optical microscopic examination.

Additionally, penetration and dissolution behavior were monitored in 20% gelatin matrices using optical microscopy to simulate tissue interaction. A 20% (*w/v*) gelatin solution was prepared by adding a predetermined amount of gelatin powder to distilled water. After it completely dissolved under continuous stirring at 70 °C, the resulting solution was then poured into molds and allowed to cure at room temperature to obtain a visualized skin model. Subsequently, microneedle arrays were cut from the cured skin model using a doctor blade. Both the microneedle array and the skin model were fixed onto the experimental platform using double-sided tape, ensuring that the microneedle tips remained parallel to the microscope stage. The microneedle array was slowly moved toward the skin model and rapidly inserted upon contact. During the experiment, real-time images of microneedle dissolution were captured using an inverted optical microscope, and the entire penetration and dissolution process within the gelatin skin model was completely recorded.

**The transdermal penetration and retention behavior:** excised rat skin samples were mounted on Franz diffusion cells. Blank HA-MN, Cur-aggregates@MN, and Cur-PNPs@MN patches were inserted into the skin with equivalent curcumin loading. After incubation for 0.5, 2, and 6 hours, the skin was thoroughly washed with PBS, and the fluorescence of curcumin was visualized using confocal laser scanning microscopy (CLSM, Zeiss LSM 880) with excitation at 420 nm and emission at 530 nm. Skin treated with PBS served as the blank control.

### 2.5. In vitro cell experiments

#### 2.5.1. Cytotoxicity evaluation experiments

**Cytotoxicity evaluation of microneedle and nanoparticle materials:** the cytotoxicity of microneedle materials (HA-MNs) and nanoparticle carriers (Blank-PNPs) was assessed using the 3-(4,5-dimethylthiazol-2-yl)-2,5- diphenyltetrazolium bromide (MTT) assay according to ISO 10993-5. L929 and RAW264.7 cells were seeded into 96‑well plates (5 × 10^3^ cells/well) and incubated overnight. And the cells were then treated with different concentrations of HA-MN solutions and Blank-PNPs for 24 hours. After the treatment period, 20 μL of MTT solution (5 mg/mL in PBS) was added to each well and the plates were incubated for an additional 4 hours at 37 °C. The formazan crystals were dissolved in 150 μL of DMSO, and the absorbance was measured at 490 nm using a microplate reader. Cell viability was calculated relative to untreated control cells (set as 100%). According to ISO 10993-5, materials are considered non‑cytotoxic when cell viability remains above 70% of the negative control.

#### 2.5.2. Anti-proliferation activity

To evaluate the antiproliferative effects of Cur‑aggregates and Cur-PNPs and determine the optimal drug concentrations for subsequent sonodynamic studies, an *in vitro* assay was performed using the MTT method. L929 cells and LPS-induced RAW264.7 cells were seeded into 96-well plates and treated with varying concentrations (2–30 μg/mL) of Cur‑aggregates and Cur-PNPs for 24 hours. Cell proliferation was quantified by measuring optical density at 562 nm, and the results were used to generate antiproliferative activity curves with GraphPad Prism software to compare treatment effects and guide concentration selection for further experiments.

#### 2.5.3. Cellular uptake analysis by flow cytometry

The cellular uptake of Cur-PNPs by RAW264.7 cells was investigated using flow cytometry. RAW264.7 cells were seeded into 6-well plates at a density of 5 × 10^5^ cells per well and cultured overnight. For M1 polarization, cells were stimulated with lipopolysaccharide (LPS, 100 ng/mL) and interferon-*γ* (IFN-*γ*, 20 ng/mL) for 24 hours. Following this initial culture period, the medium was carefully removed and replaced with 2 mL of fresh complete medium containing either (1) Cur-PNPs (8 μg/mL curcumin equivalent), (2) blank nanoparticles (drug-free PNPs as negative control to assess the baseline uptake kinetics of the nanoparticles themselves), or (3) complete medium alone (blank control). Each experimental condition was prepared in triplicate. After designated incubation periods (1, 2, 4, and 8 hours) under standard culture conditions, the nanoparticle-containing medium was aspirated, and cells were washed twice with PBS. Cell detachment was achieved through trypsinization, followed by centrifugation at 1000 rpm for 5 minutes to collect the cell pellet. The harvested cells were then resuspended in 500 μL of ice-cold PBS for immediate flow cytometric analysis. The intrinsic fluorescence of curcumin was measured to determine the time-dependent cellular uptake efficiency of Cur-PNPs by RAW264.7 (M1 polarization) cells across different experimental conditions.

#### 2.5.4. Cellular uptake analysis by fluorescence microscopy

For fluorescence microscopy evaluation, laser confocal dishes were initially filled with 2 mL of culture medium with serum and pre-incubated for 30 minutes (37 °C, 5% CO₂). After pre-incubation, the medium was removed and RAW264.7 (M1 polarization) cells with high viability were seeded into the dishes at a density of 1 × 10^5^ cells per well. The dishes were immediately transferred to the incubator (37 °C, 5% CO₂) for 24 hours to allow cell adhesion. Subsequently, the medium was carefully removed and replaced with a medium free of serum containing either Cur‑aggregates or Cur-PNPs, both adjusted to 8 μg/mL curcumin equivalent concentration. Three replicate wells were prepared for each treatment group. The cells were incubated with the compounds for specified durations (1, 2, 4, and 8 hours) under standard culture conditions. These time points were selected based on the uptake kinetics of blank nanoparticles determined by flow cytometry, allowing for a comprehensive comparison of cellular internalization and intracellular distribution between Cur-PNPs and Cur‑aggregates. After the incubation, the drug-containing medium was aspirated, and the cells were washed twice with PBS buffer. Then 1  ml of tissue fixative (4% paraformaldehyde) was added to each confocal dish, and cells were fixed at room temperature for 20 minutes. The fixative was removed, and the cells were washed twice with PBS buffer. Finally, 2 ml of DAPI staining solution containing an anti - fluorescence quenching agent was added, followed by 10 minutes of staining. Cellular uptake was observed across multiple fields (at least 5 fields) of view using a laser confocal microscope.

#### 2.5.5. Evaluation of the Intracellular ROS generation ability of nanoparticles

The intracellular ROS generation capacity was assessed using RAW264.7 (M1 polarization) cells in the logarithmic growth phase, which were seeded into confocal culture dishes at a density of approximately 1 × 10^5^ cells per well and allowed to adhere overnight in a CO₂ incubator (37 °C, 5% CO₂). The cultured cells were randomly assigned to five experimental groups: (1) blank control group, (2) positive control group, (3) curcumin nanoparticle group, (4) focused ultrasound group, and (5) curcumin nanoparticle combined with focused ultrasound group. After 24 hours of initial culture, the medium was removed and replaced with the respective treatment solutions: 1 mL of serum-free DMEM containing 8 μg/mL (curcumin equivalent) Cur-PNPs for the curcumin nanoparticle group (group 3) and the curcumin nanoparticle combined with focused ultrasound group (group 5); 1 mL of serum-free DMEM for blank control group (group 1) and focused ultrasound group (group 4); 1 mL of Rosup solution (provided in the reactive oxygen species (ROS) assay kit) for positive control group (group 2). The cells were then returned to the incubator for an additional 4-hour incubation period. Following treatment, the medium was carefully aspirated and cells were washed three times with PBS to remove any residual nanoparticles or free Rosup.

For ROS detection, 500 μL of 10 μmol/L DCFH-DA solution was added to each group. Groups 4 and 5 were subsequently exposed to low-intensity focused ultrasound (LIFU) treatment at 1 W/cm^2^ power for 1 minute was selected based on established protocols in sonodynamic therapy literature and preliminary optimization (Teixeira et al. 2024; Peng et al. 2026). The 1 MHz frequency provides adequate tissue penetration, while 1 W/cm^2^ (SATA) is within the low‑intensity range that minimizes thermal effects. For *in vivo* application, the duration was extended to 5 minutes to compensate for tissue attenuation. The intracellular ROS generation was immediately visualized and quantified using confocal microscopy, with consistent imaging parameters maintained across all experimental groups to ensure comparability of results. Multiple fields (at least 5 fields) of view were examined for each sample to obtain representative data on ROS production under the different treatment conditions.

#### 2.5.6. Evaluation of in vitro therapeutic effects

The apoptotic effects of various treatments were quantitatively assessed using flow cytometry with RAW264.7 cells (M1 polarization) in their logarithmic growth phase. Cells were seeded into 6-well plates at a density of 2 × 10^5^ cells/well and cultured overnight in a carbon dioxide incubator (37 °C, 5% CO₂) until reaching approximately 80% confluence. After overnight initial culture, the medium was replaced with treatment-specific solutions according to the experimental groups: Control group, a Cur‑aggregates group, a US group, a Cur PNPs group, and a Cur PNPs + US group. The Cur concentration in each drug - containing group was 8  μg/mL. Appropriate control groups including blank control, single-staining controls, and double-staining controls were established and maintained under light-protected conditions. After 4 hours, the ultrasound group was treated with ultrasound (1 MHz, 1 W/cm^2^, 1 minutes) and then returned to the incubator for continued culture. Following a total 24-h culture period, the drug-containing medium was collected and cells were detached using 1 mL of EDTA-free trypsin, with the reaction terminated by adding back the collected medium. Cell pellets were obtained by centrifugation at 500 rpm for 5 minutes, washed twice with PBS, and gently resuspended in 0.5 mL PBS. Apoptosis and necrosis rates were determined using an Annexin V-APC/7-AAD double-staining apoptosis detection kit, with flow cytometric analysis performed to quantify the differential apoptotic responses across treatment groups.

#### 2.5.7. Evaluation of macrophage polarization markers

To elucidate whether the reduction in inflammatory markers resulted from functional suppression or physical cell clearance, we performed a combined (enzyme-linked immunosorbent assay) ELISA and Cell Counting Kit-8 (CCK-8) assay. RAW264.7 cells were seeded into 6-well plates at a density of 5 × 10^5^ cells per well and cultured overnight. For M1 polarization, cells were stimulated with lipopolysaccharide (LPS, 100 ng/mL) and interferon-*γ* (IFN-*γ*, 20 ng/mL) for 24 hours. For M2 polarization, cells were stimulated with interleukin-4 (IL-4, 20 ng/mL) and interleukin-13 (IL-13, 20 ng/mL) for 24 hours.

Following stimulation, the cells were treated with Cur‑aggregates, Cur-PNPs (8 μg/mL curcumin equivalent), and/or low-intensity focused ultrasound (1 MHz, 1 W/cm^2^, 1 minute) as described in Section 2.5.6. After further incubation for 24 hours, the culture supernatants were collected and centrifuged to remove debris. The remaining adherent cells were washed twice with PBS, and 10 μL of CCK-8 solution (Dojindo, Kumamoto, Japan) was added to each well. After incubation at 37 °C for 2 hours, the absorbance was recorded at 450 nm (OD₄₅₀, CCK-8) to quantify the relative number of viable cells.

The levels of M1-associated tumor necrosis factor-*α* (TNF-*α*) and M2-associated interleukin-10 (IL-10) in the supernatants were determined using commercial ELISA kits (R&D Systems), with the optical density measured at 450 nm (OD₄₅₀, ELISA). All experiments were performed in triplicate.

#### 2.5.8. Evaluation of ultrasound-induced thermal effects

To exclude the possibility that ultrasound-induced heating contributed to the therapeutic outcomes, real-time surface temperature changes were monitored using a high-resolution infrared thermal imaging camera (P20 max V2, HIKMICRO, China). The camera was positioned perpendicular to the sample surface at a fixed distance of 10 cm to ensure full coverage of the insonated area. All measurements were performed on excised porcine skin samples (approximately 2 × 2 cm^2^, thickness ≈ 2 mm). Two groups were evaluated: (1) ultrasound group (1 MHz, 1 W/cm^2^, SATA, 5 minutes); (2) control group (no ultrasound) and temperature was recorded at 1-minute intervals.

### 2.6. Establishment and treatment of acute gouty arthritis (AGA)-bearing rats

Forty-eight male SD rats were randomly allocated into eight groups (*n* = 6 per group) by blindly picking animals from cages: (1) control group (normal saline injection), (2) model group (MSU-induced arthritis without treatment), (3) US group (ultrasound treatment only), (4) positive control group (colchicine 0.3 mg/kg by gavage), (5) blank microneedle group (HA-MN), (6) Cur‑aggregates microneedle group (Cur‑aggregates @MN), (7) SDT combination therapy group (Cur-PNPs@MN + US), (8) Cur-PNPs microneedle group (Cur-PNPs@MN). In this study, the individual animal was considered the experimental unit. The sample size (*n* = 6 per group, 48 rats in total) was determined based on preliminary experiments, which showed that this number was sufficient to achieve a statistical power of 80% with a significance level of 0.05. All treatment groups received continuous intervention for 5 days, except the control and model groups which remained untreated. The sonodynamic treatment conditions was 1 MHz and 1 W/cm^2^ for 5 min and the dose of Cur-PNPs used in the *in vivo* experiments were 3.0 mg/day. Rats were included in the study if they successfully received the intra-articular injection without signs of perforating injury. Exclusion criteria were predefined as: (1) failure to develop joint swelling 24 hours post-MSU injection; (2) death during the experimental period. All 48 rats completed the study and were included in the final analysis.

Prior to *in vivo* experiments, all rats were anesthetized with isoflurane to minimize suffering during procedures. The acute gouty arthritis (AGA) model was established by intra-articular injection of 0.2 mL of monosodium urate (MSU) suspension (25 mg/mL) into the left ankle joint of rats in both model and treatment groups. For injection, the left ankle joint was positioned at 90° of flexion, and a 1 mL syringe with a No. 3 needle was inserted perpendicularly into the posterolateral aspect of the joint; successful injection was confirmed by the appearance of a distinct bulge on the contralateral side of the joint capsule without drug leakage. The control group received an equivalent volume of sterile normal saline administered *via* the same procedure. Joint swelling was quantitatively assessed using a standardized ligature method: with the ankle fixed at 180° flexion, a marked horizontal line was used as a reference point for circumference measurements taken pre-injection and at 1, 2, 3, 4, and 5 days post-induction using inelastic string and vernier calipers, with swelling rates calculated accordingly. The primary outcome measure for evaluating therapeutic efficacy was the reduction in joint swelling rate on day 5. Secondary outcome measures included histological scores and serum cytokine levels.

For histopathological analysis, harvested ankle tissues were fixed in 4% paraformaldehyde, decalcified in 10% EDTA for 4 weeks, and processed into 4-μm paraffin sections for hematoxylin-eosin staining and microscopic evaluation. Concurrently, serum levels of pro-inflammatory cytokines (TNF-*α*, IL-6, IL-1β) were quantified using commercial ELISA kits according to manufacturer protocols to assess systemic inflammatory responses across treatment groups.

To reduce assessment bias, all *in vivo* experiments were performed in a blinded manner. For treatment administration, animals were randomly grabbed from their cages. To minimize cage location effects, cages were systematically rotated daily on the racks. The researchers who administered the treatments were different from those who performed the outcome assessments (joint swelling measurement, histological evaluation, and ELISA). Treatment allocation was concealed from the assessors until all data were collected and analyzed.

### 2.7. Statistical analysis

All data are expressed as mean ±  standard deviation (SD). Significant differences were derived by Student's t-test using Graph Pad. For comparisons among multiple groups, one-way or two-way analysis of variance (ANOVA) was used, followed by Tukey's post-hoc test for multiple comparisons. For comparisons between two groups, an unpaired two-tailed Student's t-test was used. If the data violated normality or equal variance assumptions, non‑parametric tests were applied: the Mann–Whitney U test for two groups, and the Kruskal–Wallis test with Dunn's post‑hoc test for multiple groups. *p < 0.05, **p < 0.01, ***p < 0.001, and ****p < 0.0001 were considered statistically significant; ns denotes no significant difference. All statistical analyzes were performed using GraphPad Prism 10.0.

## 3. Results and discussion

### 3.1. Characterization of PCADK

The ^1^H nuclear magnetic resonance (NMR) spectrum of PCADK is shown in Figure 3A. and displayed the expected macromolecular structure of the polymer, with the following chemical shift assignments. The peak corresponding to -CH_2_- connected to the oxygen atom of the ketal bond is located at *δ* = 3.18–3.20 ppm. The peak corresponding to -CH- on the cyclohexane ring is at *δ* = 1.84. The peaks corresponding to -CH_2_- on the cyclohexane ring are at *δ* = 0.93–1.54. The peak corresponding to -CH_3_- connected to the carbon atom of the ketal bond is at *δ* = 1.32. The Fourier transform infrared (FT-IR) spectrum of PCADK is shown in Figure 3B with the following vibrational assignments: The C—H stretching peaks of methylene and methyl groups are in the range of 2800–2960 cm^−1^. The deformation vibration peak of the methylene group is at 1450 cm^−1^. Finally, the absorption peak of the ketal bond ─C─O─C─ is at 1040  cm^−1^. The above results are highly consistent with those in the literature, indicating that PCADK has been successfully synthesized (Sun et al. 2021). The X-ray diffraction (XRD) pattern of PCADK is shown in Figure 3C (Sun et al. 2021). The characteristic peaks of PCADK appear at 12.82° and 18.14°, indicating that the synthesized PCADK exists partially in a crystalline state.

**Figure 3.**
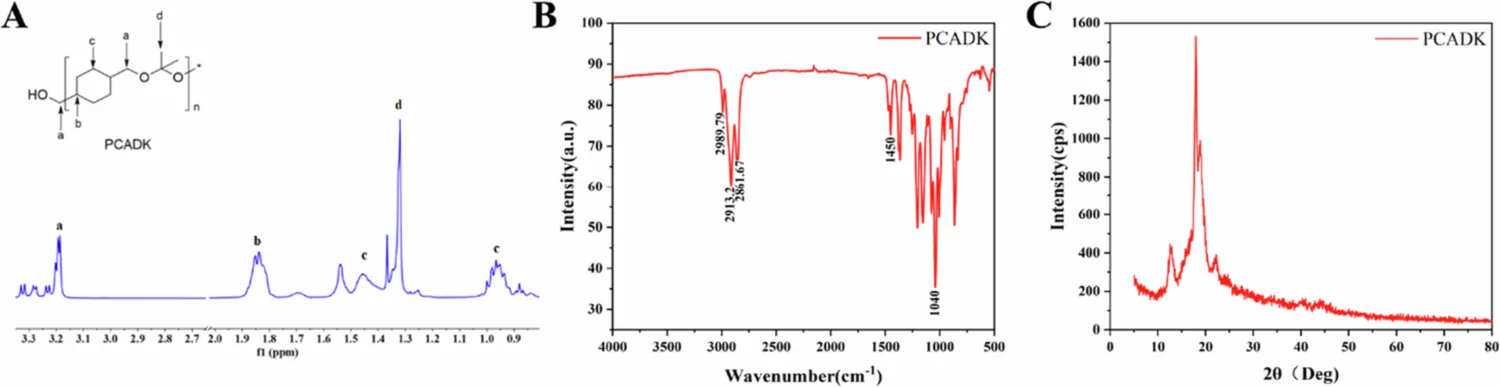
Characterization of PCADK. (A) 1 H NMR spectrum of PCADK. (B) FT-IR spectrum of PCADK. (C) XRD pattern of PCADK.

### 3.2. Characterization of Cur-PNPs

Cur-PNPs were successfully prepared by the emulsion solvent evaporation method. We optimized various parameters, including the mixed solvents ratio, surfactant concentration, and drug concentration, to determine the optimal preparation conditions as follows: a dichloromethane-to-acetone ratio of 2:1, 2% PVA as the aqueous phase for the primary emulsion, and a drug concentration of 2 mg/ml. The EE% and DLC% of the Cur-PNPs were 88.11% ± 1.37% and 4.17% ± 0.01% (the high reproducibility of the EE values across three independent batches indirectly confirms the adequacy of this sedimentation-based separation). As shown in Figure 4A, the average particle size of Cur-PNPs measured by dynamic light scattering(DLS) using Malvern particle size analyzer was 236.2 ± 1.6 nm, with a PDI of 0.115 ± 0.01. The size distribution profile showed a single peak, indicating a narrow size distribution. The morphology of Cur-PNPs observed under transmission electron microscopy (TEM) and scanning electron microscopy (SEM) is shown in Figure 4B. The nanoparticles exhibited a spherical shape, uniform distribution, and an average size of approximately 200 nm. The smaller size observed by TEM and SEM compared to DLS measurements is attributed to the different principles of the two techniques: DLS measures the hydrodynamic diameter including the hydration layer in aqueous solution, whereas TEM and SEM visualize the dried nanoparticle core. Figure 4C displays the X-ray diffraction (XRD) patterns of Cur‑aggregates (Cur), blank nanoparticles (Blank-PNPs), and Cur-PNPs. The XRD profile of Cur showed numerous sharp and intense diffraction peaks, indicating that the drug is highly crystalline. In contrast, the characteristic diffraction peaks of curcumin disappeared in the Cur-PNPs sample, suggesting that the drug transitioned from a crystalline to an amorphous solid dispersion and was uniformly dispersed within the PCADK nanoparticles. Moreover, the diffraction pattern of Cur-PNPs was similar to that of Blank-PNPs, confirming that curcumin was successfully encapsulated within the nanoparticles rather than merely adsorbed on the surface. The stability of the nanoparticles is illustrated in Figure 4D. These results demonstrate that under both storage conditions, the particle size remained around 240 nm without significant change, indicating no aggregation. The PDI values remained below 0.2 throughout the study, reflecting good dispersion stability. These results confirm that Cur-PNPs remain stable for at least half a month under the tested conditions.

**Figure 4.**
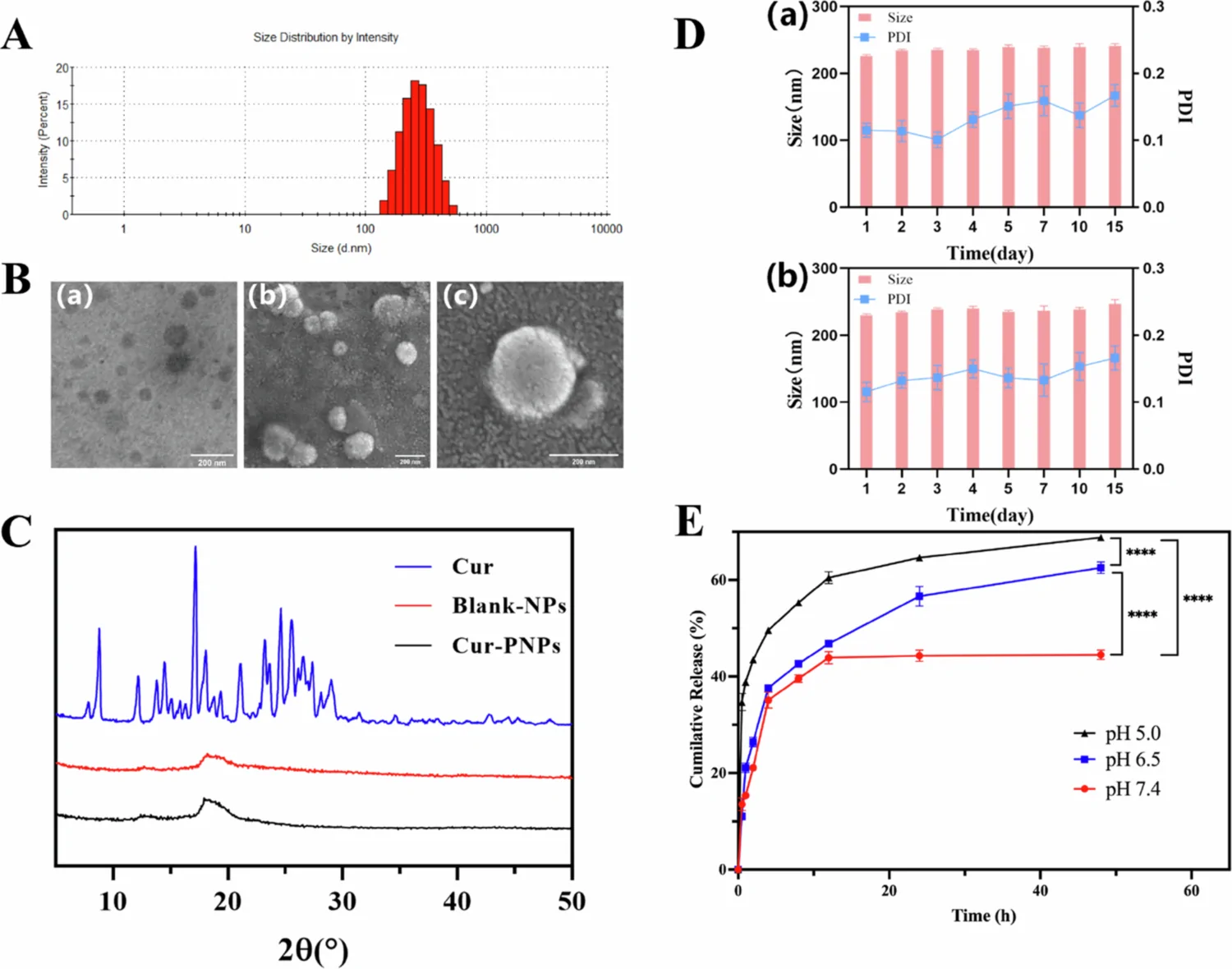
Characterization of Cur-PNPs. (A) Size of Cur-PNPs. (B) Image of Cur-PNPs (a: TEM image; b, c: SEM image). (C) X-ray powder diffraction patterns of Cur, Blank-PNPs, and Cur-PNPs. (D) Stability map of Cur-PNPs within 15 days (a: 4 ± 2 °C; b: 28 ± 2 °C). (E) *In vitro* drug release profile of Cur-PNPs at pH 5.0, pH 6.5, and pH 7.4 (*n* = 3). Statistical analysis was performed using two-way ANOVA with Tukey's post-hoc test.

### 3.3. pH-responsive drug release behavior of Cur-PNPs in vitro

PCADK exhibits pH-responsive characteristics and maintains structural integrity under neutral conditions. However, under acidic environments (Feng Pu et al., 2015), the ketal bonds within its molecular structure undergo selective cleavage, leading to material hydrolysis and subsequent release of encapsulated therapeutic agents. To investigate the acid-sensitive release behavior of Cur-PNPs, we performed *in vitro* release studies using the dialysis bag method in PBS at pH 7.4 (simulating normal physiological conditions), pH 6.5 (mildly acidic inflammatory microenvironment of AGA), and pH 5.0 (simulating the strongly acidic extreme to demonstrate the robust acid-responsiveness of the PCADK polymer) (Yu et al. 2019).

As shown in Figure 4E, there was a significant burst release of the drug within 0–30 minutes across all pH conditions, which can be primarily attributed to the dispersion of drug molecules adsorbed on the surface or in the vicinity of the nanoparticles. Notably, the drug release exhibited a clear pH-dependent profile throughout the 48 hours observation period. At pH 7.4, the cumulative release reached 44.30% ± 1.96% at 24 hours and remained relatively stable at 44.51% ± 1.69% at 48 hours, indicating that drug release at physiological pH occurs primarily through passive diffusion with minimal polymer degradation.

In contrast, at pH 6.5 (closely approximates the mild acidification characteristic of the inflamed joint microenvironment in AGA) the release increased substantially to 56.63% ± 3.51% at 24 hours and further to 62.54% ± 2.07% at 48 hours. This significantly enhanced release at a pathologically relevant pH confirms that Cur-PNPs can effectively respond to the acidic conditions at the disease site, delivering curcumin precisely where it is needed. At pH 5.0, the cumulative release reached 64.63% ± 1.25% at 24 hours and 68.81% ± 1.14% at 48 hours, reflecting the most pronounced polymer degradation.

The progressive enhancement of drug release with decreasing pH (7.4 < 6.5 < 5.0) demonstrates that Cur-PNPs are capable of responding to a range of acidic stimuli relevant to the inflammatory microenvironment. Most importantly, the substantial release at pH 6.5 ensures that therapeutic concentrations of curcumin can be achieved directly at the pathological site while minimizing premature release under normal physiological conditions, aligning with the microenvironment-targeting design of our system.

### 3.4. Investigation of the in vitro ROS generation ability of nanoparticles

The ability of the nanoformulation to generate ROS, in particular singlet oxygen (^1^O_2_) was thoroughly examined in order to ascertain the stability and sonodynamic activity improvements of curcumin. ROS generation was quantified using specific molecular probes: DPBF (427 nm) for general ROS (including ^1^O₂, •OH, and O₂•^-^) and ABDA (400 nm) for singlet oxygen (^1^O₂), where the progressive decrease in absorbance directly correlates with increased ROS production through the probe oxidation. As shown in Figure 5A, ultrasound-activated Cur-PNPs and Cur‑aggregates exhibited significantly greater ABDA absorbance reduction compared to control group. These results indicate that curcumin has a strong ultrasound-activated ^1^O₂ generation capacity. The improved performance was further quantified through comprehensive ROS analysis using DPBF. As shown in Figure 5B, the absorbance in the Cur-PNPs group decreased more than Cur‑aggregates group, which indicates that nanoparticle encapsulation can produce general ROS more efficiently. In addition, we investigated the factors affecting the sonodynamic activity by changing the concentration of Cur-PNPs, sonication time, and ultrasonic power. The relationship between the nanoparticle concentration and sonodynamic activity is shown in Figure 5C. As the concentration of Cur-PNPs increases, the absorbance of DPBF gradually decreases, proving that the production of general ROS increases. The influence of ultrasonic power on the sonodynamic activity is shown in Figure 5D. As the ultrasonic power increases, the absorbance of DPBF also gradually decreases. Combining with the change of the full-wavelength UV-Vis curve of DPBF with ultrasonic irradiation time in Figure 5E, it can be shown that the sonodynamic activity of Cur-PNPs is positively correlated with the nanoparticle concentration, ultrasonic power, and sonication time.

To further investigate the specific effects of nanoencapsulation on curcumin's sonodynamic activity, we conducted full-wavelength spectral scanning analyzes. As shown in Figure 5E, the time-dependent decrease in absorbance at 427 nm observed corresponds precisely to the characteristic absorption wavelength of DPBF. The spectral specificity of this decay with no significant changes observed at other wavelengths, confirming effective general ROS generation through ultrasound-activated Cur-PNPs. Combining with the change of the full - wavelength UV-Vis curve of ABDA with Figure 5F, the Cur‑aggregates group exhibited pronounced time-dependent absorbance decay in the 430–500 nm range, was the stability of curcumin's intrinsic spectral features (particularly at ~430 nm) throughout sonication further supports that the observed activity originates from carrier degradation rather than enhanced sonodynamic performance. Distinct from DPBF's characteristic 427 nm absorption, this spectral signature clearly indicates auto-degradation of non-encapsulated curcumin under sonication. In contrast, Cur-PNPs maintained stable absorption in this region, demonstrating preservation of curcumin's structure.

This provides direct evidence that nanoencapsulation significantly boosts curcumin's ROS-generating capability while maintaining molecular integrity under ultrasonic conditions. This improved performance can be attributed to the nanoparticle's dual function of protecting the sonosensitizer from environmental degradation while simultaneously enhancing its energy transfer efficiency during acoustic activation.

**Figure 5.**
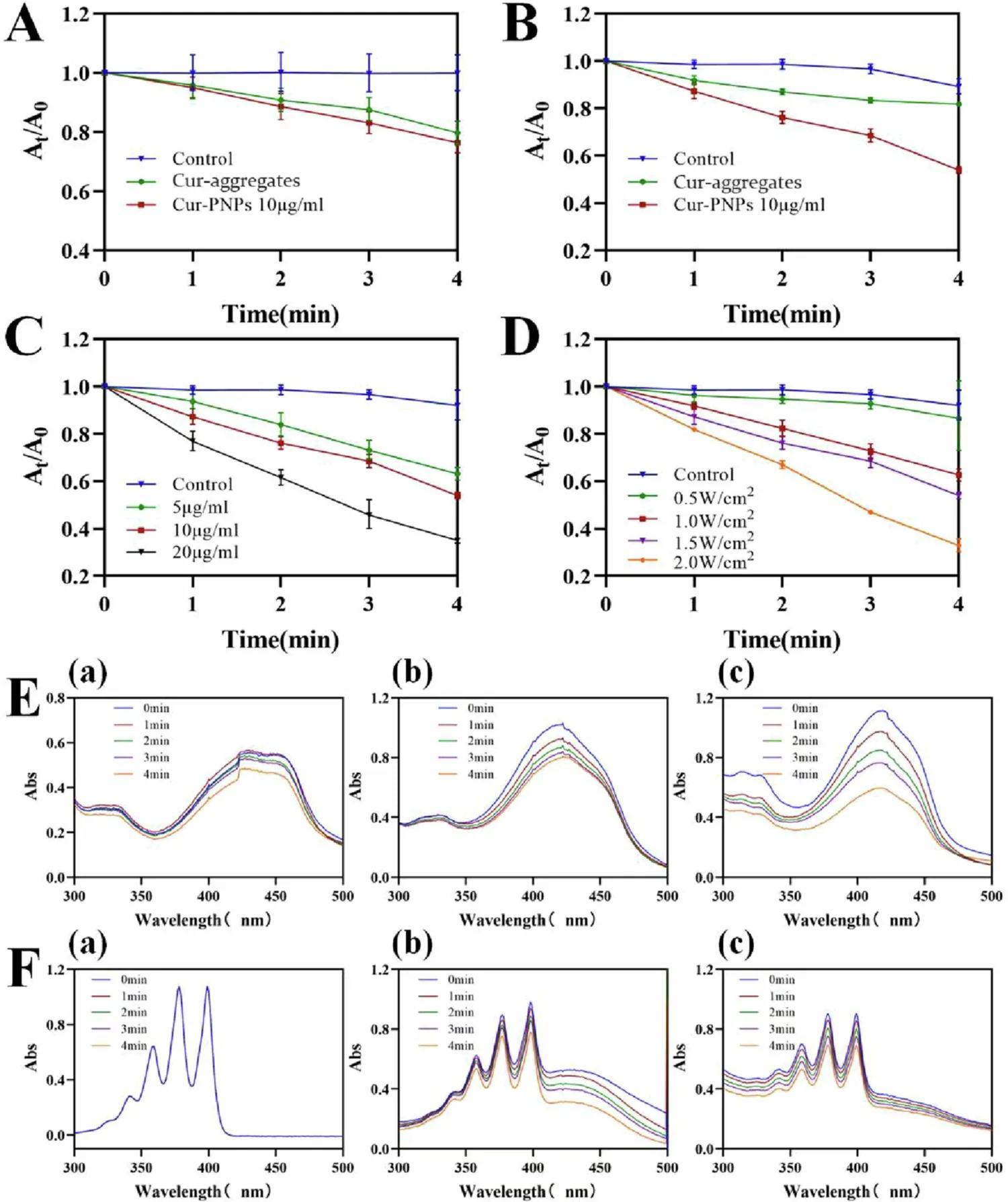
UV-vis absorbance change curve of DPBF (ROS) or ABDA (^1^O₂) treatment. (A) Absorbance change curves of different samples using ABDA as a probe (*n* = 3). (B) Absorbance change curves of different samples using DPBF as a probe (*n* = 3). (C) Absorbance change curves of samples with different concentrations using DPBF as a probe (*n* = 3) (10  mL of Cur-PNPs nanoparticle suspensions with concentrations of 5, 10 and 20 μg/mL, respectively). (D) Absorbance change curves of samples under different acoustic intensities using DPBF as a probe (*n* = 3) (10 μg/mL Cur-PNPs nanoparticle suspension). (E) UV-vis absorption spectra of different samples as a function of sonication time using DPBF as a probe (a: Control; b: Cur‑aggregates; c: Cur-PNPs). (F) UV-vis absorption spectra of different samples as a function of sonication time using ABDA as a probe (a: Control; b: Cur‑aggregates; c: Cur-PNPs). Statistical analysis was performed using two-way ANOVA with Tukey's post-hoc test.

### 3.5. Characterization of Cur-PNPs@MN

#### 3.5.1. Morphological characterization of microneedles

The photographs of the microneedle arrays are shown in Figure 6A (a) and (b) are the blank microneedles and drug - loaded microneedles, respectively. The overall shape is a 1 cm × 1 cm square, and each piece of MNs is arranged in a grid of 10 × 10 array. Figure 6A(c) shows the lateral morphology of a row of microneedles under an optical microscope. At higher resolution, Figure 6A (d) and (e) display the morphology of the microneedles revealed by SEM. Each needle presents an approximately conical shape, with uniform shape and size. The needle length is 758.6 ± 1.2 μm, and the diameter at the bottom of the needle is 312.6 ± 0.7 μm. Therefore, the hyaluronic acid microneedles (HA-MNs) prepared by the one-step method have sharp tips, smooth needle surfaces, identical shapes, consistent lengths, and good uniformity.

#### 3.5.2. Mechanical strength and skin penetration ability of microneedles

After preparing the hyaluronic acid microneedles (HA-MN), we evaluated their mechanical properties. As shown by the force–displacement curve (Figure 6C), the HA-MN array reached a critical buckling force of approximately 5.70 *N*, corresponding to 0.633 *N* per needle. In comparison, the force required to penetrate the skin is 3.596 × 10^−4^ *N* (Yan et al. 2021). Thus, the mechanical strength of the microneedle tips is sufficient to penetrate the skin. To further verify the mechanical properties of the HA-MN, weights were applied to the tips of the microneedles to visually evaluate their bending resistance. As shown in Figure 6D, when the force applied on a single needle reached 0.0245 *N*, there was no obvious bending, and the compression ratio (defined as the percentage reduction in needle height measured from optical microscopy images) was only 10.8%, indicating that the needles were not damaged. When the force increased to 0.1125 *N* per needle, obvious bending began to occur at the tip, accompanied by a compression ratio close to 20%, marking the onset of mechanical failure. Under a force of 0.49 *N* per needle, the tip exhibited significant bending with a compression ratio nearing 40%, and the mechanical properties were largely lost. Taking 0.1125 *N* as the critical point at which damage initiates, the corresponding critical force for the entire microneedle array is 11.25 *N*. This is sufficient to penetrate the skin for drug delivery without deformation and alteration of the mechanical properties of the needles.

The puncture capability of the microneedle array was further verified using trypan blue staining. Since trypan blue selectively stains dead cells, it was used to assess whether the microneedles successfully penetrated the stratum corneum and reached the intradermal tissue. As shown in Figure 6B, the pinholes left by the microneedle patch on the mouse skin surface were neat and clear, indicating that the microneedle patch had successfully penetrated the skin. The skin penetration rate of the microneedles was 100%, indicating that the microneedles had the required puncture performance and could successfully penetrate the skin.

#### 3.5.3. Dissolution performance of microneedles

The dissolution performance of the microneedles is shown in Figure 6E. After the microneedles were exposed to PBS buffer at pH 6.5 (Figure 6E(a)), the needle tips rapidly collapsed upon hydration and subsequently underwent gradual dissolution. Within 15 seconds, half of the MNs had dissolved; and the majority had dissolved after 20 seconds. To further evaluate dissolution under simulated biological conditions, the MNs were inserted into the prepared gelatin skin model (Figure 6E(b)). Prior to insertion, the microneedle tips were sharp and structurally intact. After insertion, the tips rapidly absorbed moisture from the gelatin, leading to initial structural collapse followed by progressive dissolution. Complete dissolution was achieved within 40 seconds. These results demonstrate that the HA-MNs have suitable dissolution characteristics for the envisioned application. Upon skin penetration, they could efficiently absorb interstitial moisture, dissolve rapidly, inducing a prompt release of the encapsulated drug.

#### 3.5.4. *Ex vivo* Investigation of the penetration depth of microneedles into the skin

To evaluate the feasibility of transdermal drug delivery of MNs at the joint site, we used a laser confocal microscope to capture cross-sectional images of the MNs after insertion into the mouse skin. These images were used to analyze the penetration depth of the MNs within the skin tissue. Figure 6F displays cross-sectional images of skin tissue from the surface (0 μm) to a depth of 300.08 μm following the application of the drug-loaded microneedles. The results indicate that the tips of the curcumin nanoparticle-loaded microneedles successfully penetrated the skin tissue, with distinct green fluorescence observed at the corresponding sites. Due to the skin's unique viscoelastic properties and non-uniform surface topology, the insertion process caused bending and deformation of the skin, leading to an actual penetration depth that is less than the microneedles' nominal height (Yuan et al. 2012). The images show that the fluorescence disappears when the section depth is 300 μm, indicating that the penetration depth of the prepared microneedles is approximately 300 μm. Thus, the microneedles are capable of penetrating the stratum corneum and reaching the epidermis and upper dermis, enabling effective transdermal delivery of nanodrugs.

**Figure 6.**
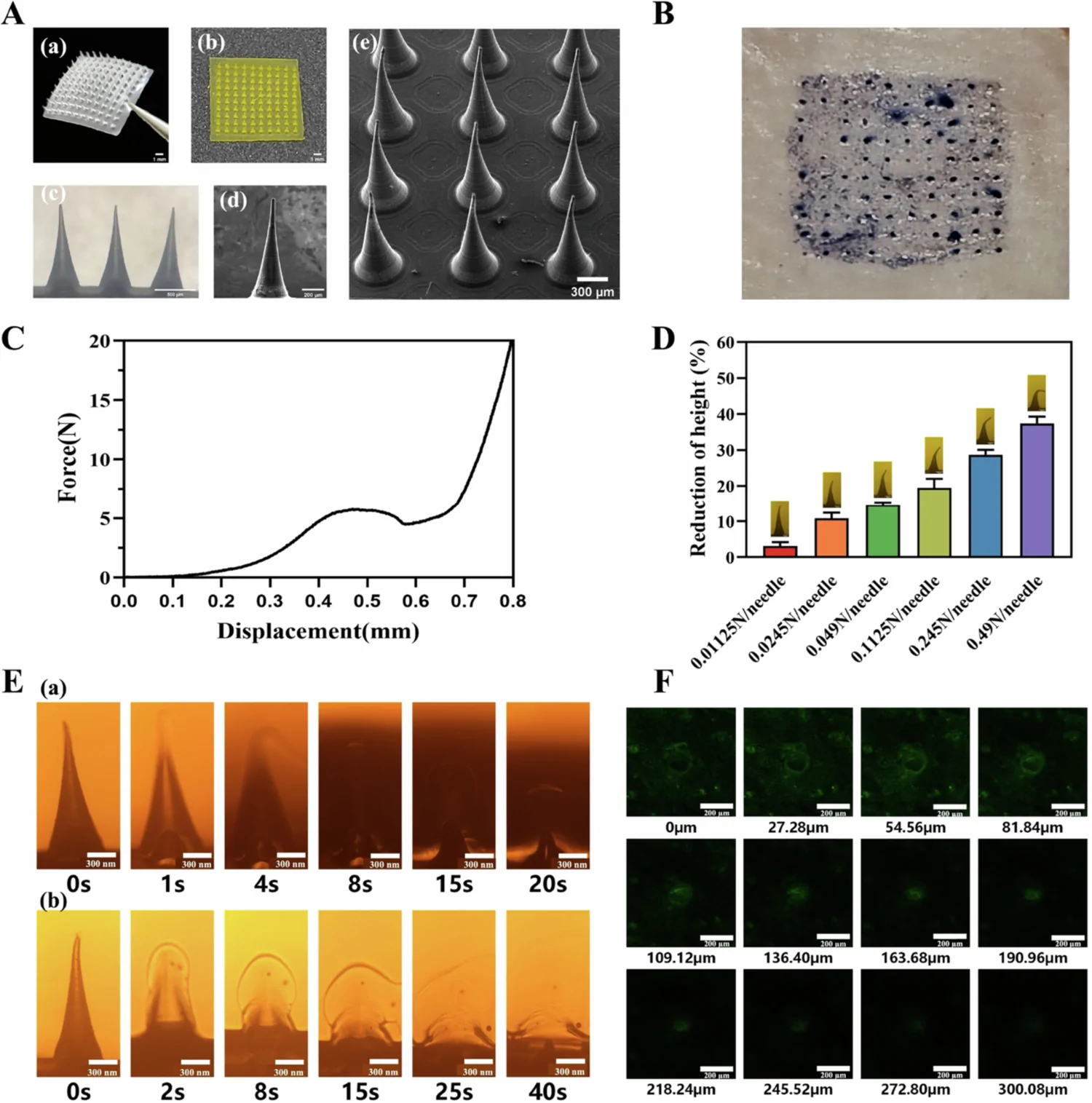
Characterization of microneedles. (A) Morphological diagram of the appearance of microneedles (a: Optical photo of blank microneedles; b: Optical photo of drug - loaded microneedles; c: Image of microneedles under an optical microscope; d, e: Scanning electron microscope (SEM) images of microneedles). (B) Trypan blue staining of mice skin after the MNs application. (C) Mechanical strength of MNs. (D) Morphological changes and compressibility of MNs under different stresses. (E) *In vitro* dissolution behavior of MNs (a: PBS; b: gelatin model). (F) Penetration depth of microchannels formed by microneedles puncturing *ex vivo* skin under confocal microscope.

#### 3.5.5. Enhanced skin penetration and retention of Cur-PNPs

Excised rat skin was used to evaluate whether the nanoparticle formulation enhances transdermal drug penetration and retention. The blank HA-MN, Cur‑aggregates@MN and Cur-PNPs@MN were inserted into the skin, and the fluorescence distribution in skin layers was visualized by confocal microscopy at 0.5, 2, and 6 hours post-application.

As shown in Figure 7, the blank HA-MN exhibited no detectable auto-fluorescence at any time point. In the Cur‑aggregates@MN group, relatively strong fluorescence was observed at the skin surface at 0.5 hours, indicating initial penetration of free curcumin. However, this fluorescence rapidly diminished by 2 hours and became barely detectable at 6 hours, suggesting rapid clearance of the free drug. In contrast, the Cur-PNPs@MN group exhibited remarkably stronger fluorescence intensity at 0.5 hours, and importantly, intense fluorescence signals remained clearly visible within the deeper dermal layers at 2 hours and persisted up to 6 hours. These results demonstrate that the Cur-PNPs@MN platform not only facilitates deeper transdermal penetration but also promotes sustained drug retention at the application site.

**Figure 7.**
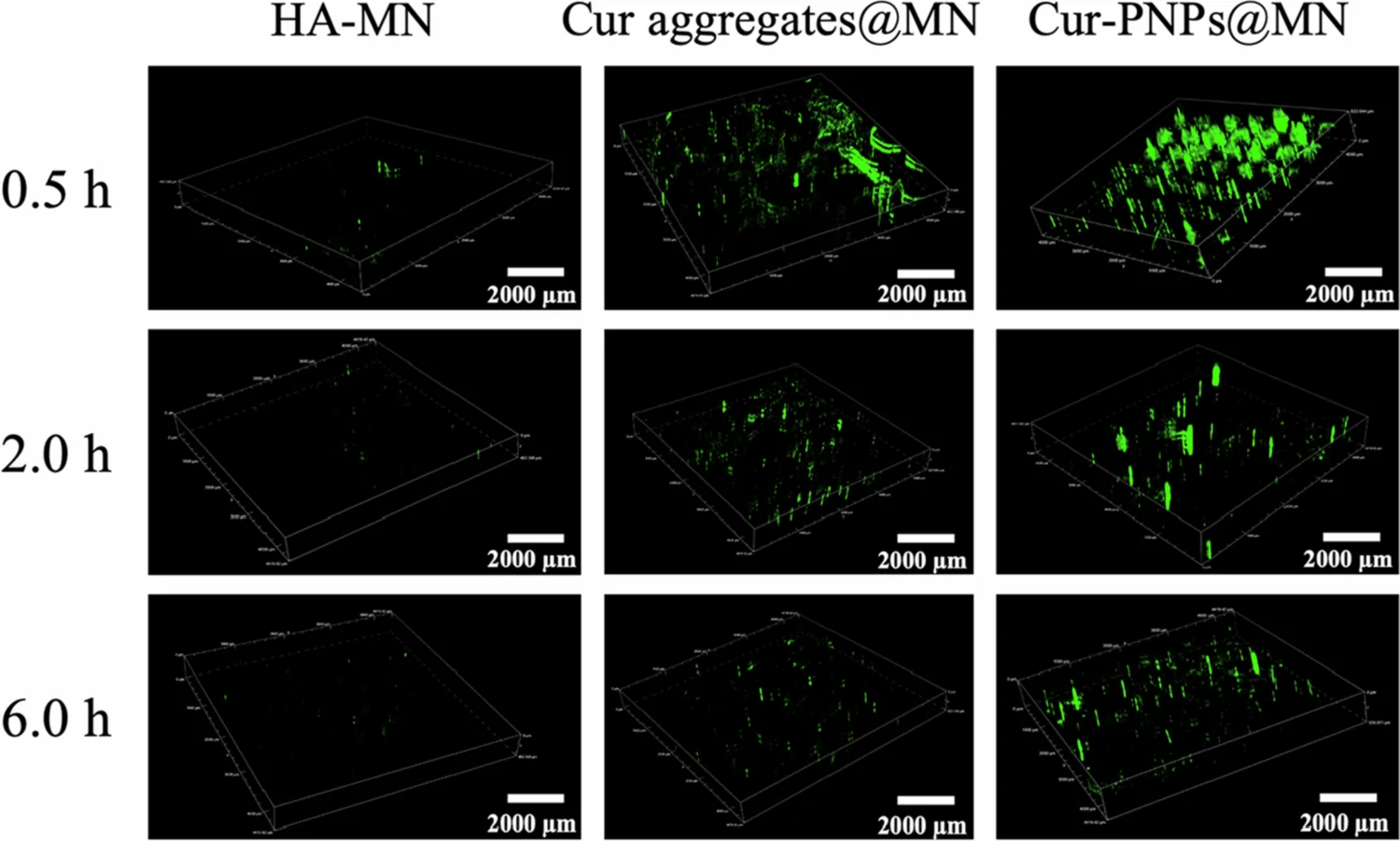
Confocal microscopy images of curcumin fluorescence in excised rat skin after insertion of Blank HA-MN, Cur-aggregates@MN, and Cur-PNPs@MN for 0.5, 2, and 6 hours.

### 3.6. In vitro cell experiments

#### 3.6.1. Investigation of the biocompatibility of materials

The cytotoxicity of the nanoparticle material (PCADK) and microneedle material (HA-MN) was evaluated as shown in Figure 8A and B. After 24 hours of co-incubation with L929 and RAW264.7 cells, both materials maintained high cell viability within the operational concentration range. The viability remained above 80% for nanoparticles and even exceeded 100% for hyaluronic acid-based microneedles, indicating negligible cytotoxicity and cytocompatibility of both carrier systems.

#### 3.6.2. Study on anti-proliferation activity in vitro

The *in vitro* anti-proliferative effects of Cur‑aggregates and Cur-PNPs on RAW264.7 and L929 cells were evaluated using the MTT assay after 24 hours of incubation to determine an effective and safe treatment concentration. As shown in Figure 8C, both Cur‑aggregates and Cur-PNPs exhibited concentration-dependent cytotoxicity. Cur‑aggregates showed a stronger anti-proliferative effect, with cell viability dropping below 80% at concentrations above 4–6 μg/mL and reaching the IC₅₀ at 8 μg/mL for RAW264.7 cells. In contrast, Cur-PNPs demonstrated significantly reduced cytotoxicity, with substantial toxicity (below 80%) only observed at 25 μg/mL. These results suggest that the nano-formulation alleviates the inherent toxicity of curcumin while allowing the application of higher drug concentrations. To minimize adverse effects and maximize the potential efficacy of subsequent SDT, a concentration of 8 μg/mL was selected for Cur-PNPs, which maintains cell viability above 85% while providing sufficient drug exposure. This approach enables the use of a therapeutically relevant dose with improved biocompatibility for further SDT applications.

#### 3.6.3. Cellular uptake of nanoparticles

To evaluate the cellular uptake efficiency of nanoparticles by macrophages, the internalization of Cur-PNPs by RAW264.7 cells was quantitatively analyzed using flow cytometry. Blank nanoparticles were first employed to establish the intrinsic internalization kinetics of the nanocarrier itself, eliminating any potential interference from the fluorescent properties or passive diffusion of Cur‑aggregates. This provided a baseline for interpreting the uptake behavior of Cur-PNPs. As shown in Figure 8D, noticeable Cur accumulation was observed within 1 hour of incubation. The uptake increased over time and reached saturation at 4 hours. These results indicate rapid and efficient phagocytosis of Cur-PNPs by murine macrophages, with the saturation point (4 hours)representing the optimal incubation time for subsequent intracellular localization studies.

To further visualize the intracellular distribution and demonstrate the advantage of nanoencapsulation over free drug delivery, confocal laser scanning microscopy was performed (Figure 8E). Based on the uptake kinetics established in Figure 8D, time points including 1, 2, 4, and 8 hours were selected for qualitative observation. In the Cur‑aggregates group, curcumin fluorescence was predominantly localized on the cell membrane with minimal cytoplasmic presence, consistent with its poor aqueous solubility and limited cellular internalization. In contrast, the Cur-PNPs group exhibited strong time-dependent fluorescence intensification within the cells. Significant intracellular accumulation was observed as early as 1 hour, and fluorescence remained stable even after 8 hours, suggesting sustained drug release from the nanoparticles after uptake. These qualitative observations not only corroborate the uptake kinetics shown in Figure 8D but also provide visual evidence for sustained drug release from the nanoparticles after internalization.

Notably, curcumin fluorescence was initially distributed in the cytoplasm and later observed in the nucleus over extended incubation periods. This phenomenon can be attributed to the acid-sensitive hydrolysis of the PCADK polymer. After cellular internalization, the nanoparticles are likely entrapped within lysosomes, where the acidic environment (pH~ 4.5 to 5.0) triggers polymer degradation and subsequent curcumin release. The liberated drug molecules then diffuse into the nucleus through nuclear pores (Wang et al. 2016).

These results show that Cur-PNPs significantly enhance macrophage uptake compared to Cur‑aggregates, which aligns with their proposed role in targeting M1-type macrophages in acidic inflammatory environments such as those found in AGA. The efficient and sustained intracellular delivery supported by Cur-PNPs provides a rational basis for their use in sonodynamic therapy, where adequate substrate accumulation within target cells is essential for ROS generation under ultrasound irradiation.

**Figure 8.**
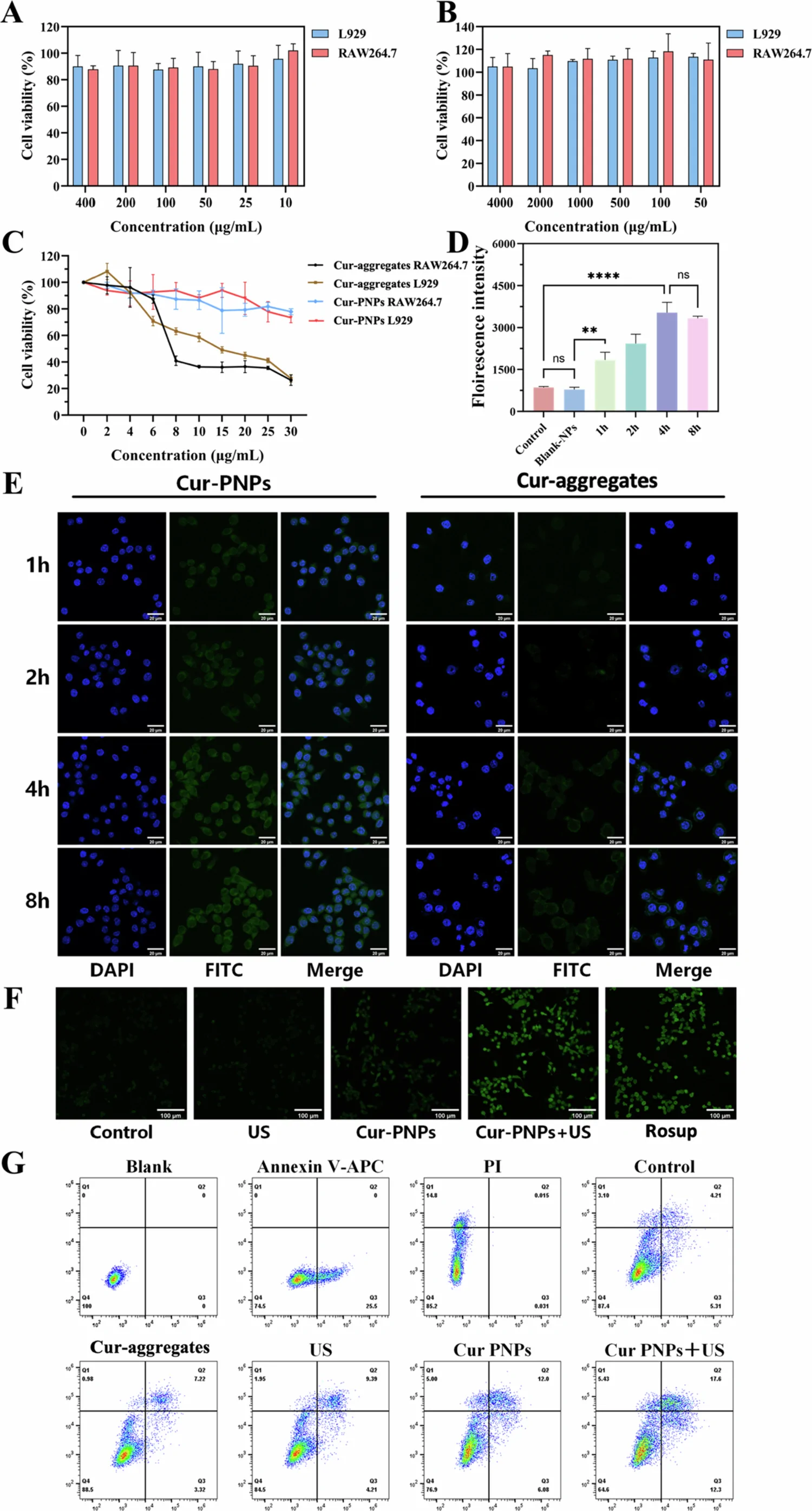
Results of cell experiments. (A) Cytotoxicity of blank nanoparticles (*n* = 6). (B) Cytotoxicity of microneedle materials (*n* = 6). (C) *In vitro* anti - proliferative capabilities of Cur‑aggregates and Cur-PNPs (*n* = 6). (D) *In vitro* RAW264.7 cell uptake of Blank-PNPs and Cur-PNPs during incubation. (E) Fluorescence microscopy images of cellular uptake of Cur-PNPs and Cur‑aggregates in RAW264.7 cells. (F) Confocal images of intracellular reactive oxygen species generation ability. (G) Apoptosis results of RAW264.7 cells after different treatments. Statistical analysis was performed using two-way ANOVA with Tukey's post-hoc test.

### 3.7. Evaluation of the intracellular ROS generation ability of nanoparticles

In this study, intracellular ROS levels in different treatment groups were assessed using the DCFH-DA probe in conjunction with fluorescence microscopy. The non-fluorescent DCFH-DA probe enters cells and is hydrolyzed by cellular esterases to DCFH, which accumulates intracellularly. Upon oxidation by reactive oxygen species, DCFH is converted to the fluorescent compound DCF, which emits green fluorescence at 488 nm excitation, with its intensity directly proportional to the amount of ROS produced. As shown in Figure 8F, both the Control and US-only groups showed negligible green fluorescence, indicating that focused ultrasound alone does not elicit significant ROS production. A mild increase in fluorescence was observed in the Cur-PNPs group, which may be attributed to the spectral overlap between the autofluorescence of internalized curcumin and the DCF emission signal. In contrast, the group treated with Cur-PNPs combined with focused ultrasound displayed strong green fluorescence, similar to that of the positive control. These results clearly demonstrates that curcumin, when delivered via PCADK nanoparticles and activated by low-frequency focused ultrasound, effectively induces substantial intracellular ROS generation.

### 3.8. Evaluation of in vitro therapeutic effects

The *in vitro* therapeutic effects of different treatment on LPS‑induced RAW 264.7 macrophages (M1‑like phenotype) are shown in Figure 8G. At a relatively low concentration, Cur‑aggregates induced an apoptotic rate of only 10.54%, marginally higher than the 9.52% observed in the Control group. This minimal pro-apoptotic effect suggests that Cur‑aggregates has limited ability to induce cellular apoptosis, which can be attributed to its inherently poor solubility and low cellular uptake efficiency, as preliminarily indicated in the Cellular uptake assay (Figure 8E). The ultrasound-alone (US) group showed a moderate increase in apoptosis to 13.60%, which, as preliminarily indicated in the Intracellular ROS generation assay (Figure 8F). It is likely attributable to non-ROS-related factors such as thermal effects or mechanical perturbation caused by ultrasound irradiation. In contrast, the Cur-PNPs group exhibited a markedly elevated apoptotic rate of 18.08%, indicating that nanoparticle encapsulation effectively enhances cellular uptake and bioavailability of curcumin, thereby promoting its pharmacological efficacy. Most notably, the Cur-PNPs + US group demonstrated a substantial apoptotic rate of 29.9%, significantly outperforming all other groups. This pronounced effect confirms that low-frequency focused ultrasound activates curcumin as a sonosensitizer, triggering robust ROS generation and activating intrinsic apoptotic pathways. These results collectively underscore a synergistic therapeutic interaction between nanoparticle-delivered curcumin and ultrasound, establishing the efficacy of SDT in specifically inducing macrophage apoptosis—a key mechanism for the treatment of inflammatory diseases such as AGA.

### 3.9. Effects of treatments on cytokine secretion in polarized macrophages

The secretion of M1-associated TNF-*α* and M2-associated IL-10 were evaluated via ELISA. As shown in Figure 9, LPS/IFN-*γ* stimulation robustly upregulated TNF-*α* from 0.179 ± 0.008 (Blank) to 2.200 ± 0.096 (M1 Control), confirming successful M1 polarization, while IL-4 stimulation significantly elevated IL-10 from 0.170 ± 0.015 to 0.633 ± 0.109 (M2 Control), confirming successful M2 induction.

Among all treatments, the SDT group (Cur-PNPs + US) exhibited the most pronounced reduction in TNF-*α*, decreasing to 1.415 ± 0.066, representing a 35.7% decrease from the control group. In contrast, Cur‑aggregates showed no significant effect (2.135 ± 0.151), while Cur-PNPs alone (1.788 ± 0.064) and US alone (1.824 ± 0.113) exhibited moderate but statistically significant reductions, confirming the synergistic enhancement upon combination therapy. Notably, when evaluating the effects on M2 macrophages, none of the treatments caused a statistically significant change in IL-10 secretion compared to the M2 control group, with the SDT group showing 0.695 ± 0.091. This clearly demonstrates the SDT preferentially reduced M1-associated TNF-*α* secretion, whereas IL-10 secretion from M2-polarized cells was not significantly changed under the present conditions.

Remarkably, the 35.7% reduction in TNF-*α* secretion closely parallels the 29.9% apoptotic rate observed in M1 macrophages upon SDT treatment (Figure 8G). This quantitative correlation strongly suggests that the primary mechanism of action is the physical elimination (apoptotic clearance) of pathogenic M1 macrophages, rather than global transcriptional repression of the entire population. Meanwhile, the moderate inhibition observed in the Cur-PNPs alone group (18.7% reduction) reflects the intrinsic anti-inflammatory activity of curcumin on the residual viable cells, serving as a complementary but secondary contribution. Collectively, these data establish that the chemo-sonodynamic synergy predominantly operates through selective cytoreduction of M1 macrophages, while sparing the M2 anti-inflammatory population, thereby providing a mechanistic basis for the superior therapeutic outcomes observed *in vivo*.

**Figure 9.**
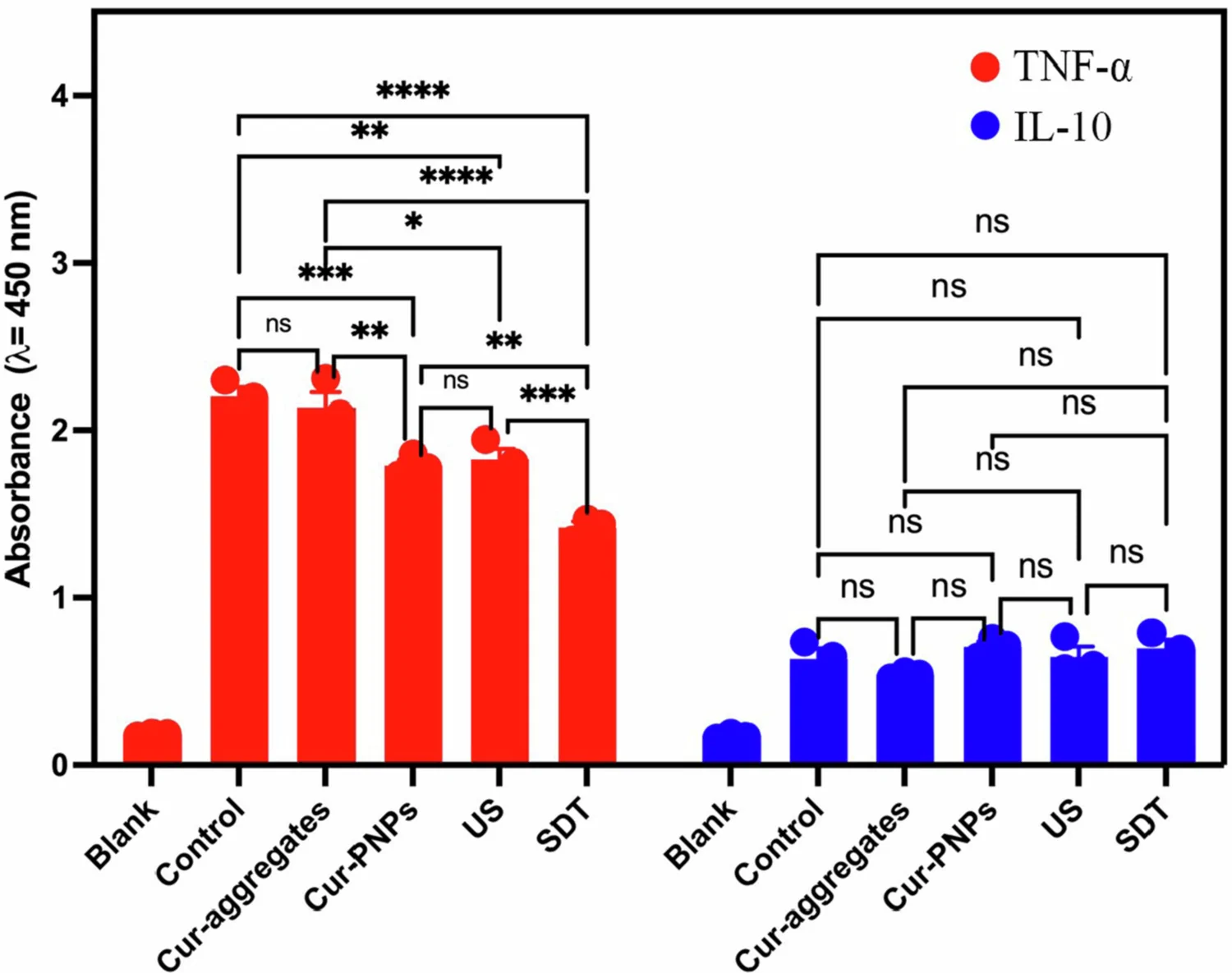
Effects of different treatments on TNF-*α* and IL-10 secretion in polarized RAW264.7 macrophages (*n* = 3). Statistical analysis was performed using two-way ANOVA with Tukey's post-hoc test.

### 3.10. Evaluation of ultrasound-induced thermal effects

To exclude the possibility of ultrasound-induced thermal effects, we monitored the surface temperature of excised porcine skin during sonication using infrared thermography. As shown in Figure 10, no significant temperature elevation was observed in either the ultrasound group or the control group throughout the 5-minute observation period. At each time point (1, 2, 3, 4, and 5 minutes), there was no statistically significant difference in surface temperature between the two groups. These results confirm that thermal effects were negligible under our experimental conditions (1 MHz, 1 W/cm^2^, 5 minutes) and that the therapeutic outcomes observed in the Cur-PNPs + US group are predominantly attributable to sonodynamic ROS generation rather than thermal ablation or hyperthermia.

**Figure 10.**
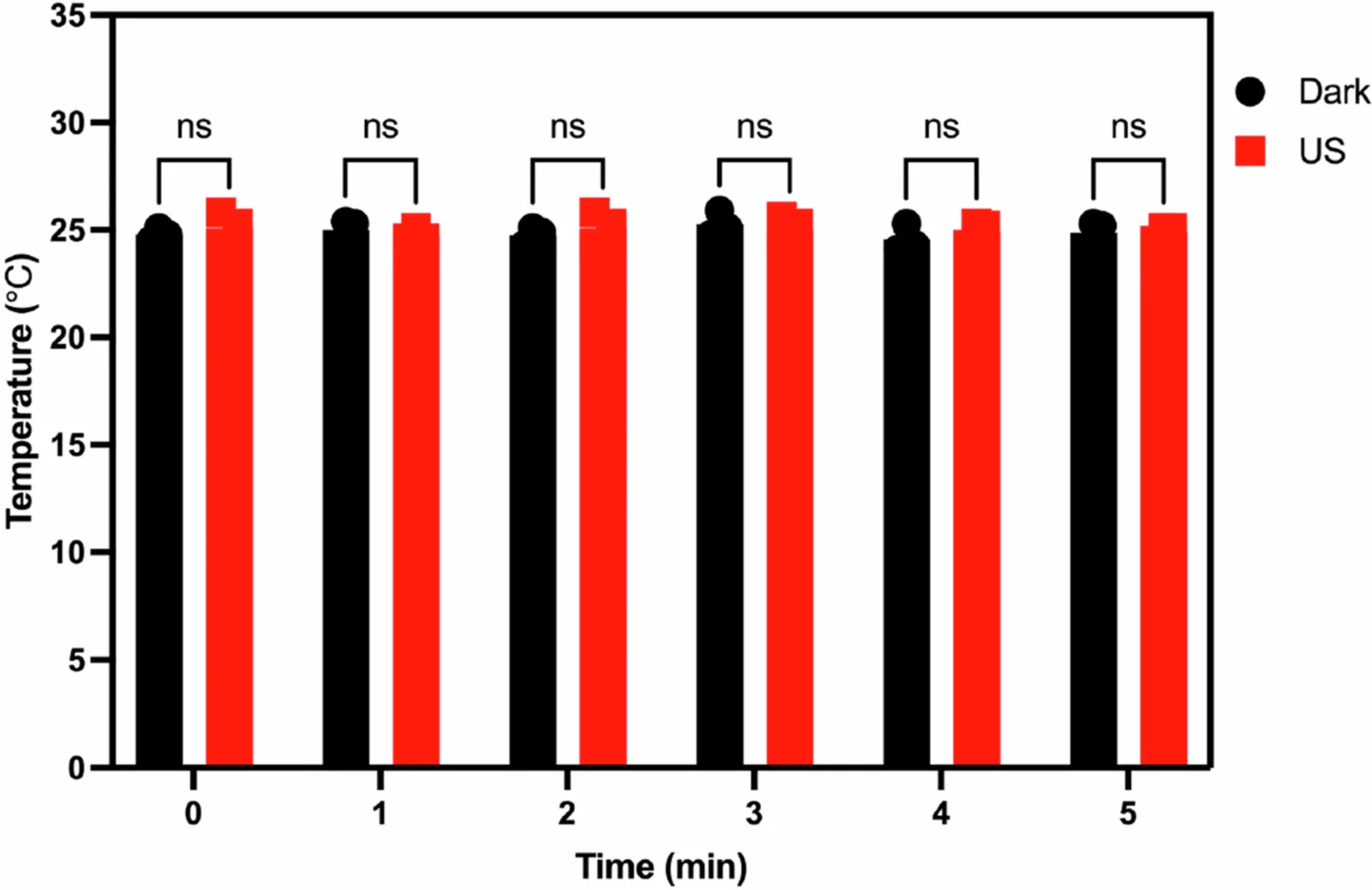
Infrared thermal imaging evaluation of ultrasound-induced thermal effects on excised porcine skin. Statistical analysis was performed using two-way ANOVA with Tukey's post-hoc test.

### 3.11. The therapeutic effect of Cur-PNPs@MN combined with SDT on acute gouty arthritis

#### 3.11.1. Evaluation of joint swelling rate

To evaluate the anti-inflammatory efficacy of SDT *in vivo*, a rat model of AGA was established by intra-articular injection of monosodium urate (MSU) crystals, with saline injection serving as the control. As illustrated in Figure 11A, pronounced ankle swelling was observed in MSU-treated rats, closely resembling the clinical manifestations of acute gout in humans.

Joint swelling was used as a key indicator to evaluate therapeutic efficacy. Macroscopic observations and ankle swelling rates were monitored in different treatment groups (Figure 11B). Treatments were initiated 24 hours post-modeling and continued for five consecutive days. The Model group exhibited sustained swelling throughout the experiment period, with the swelling rate reaching approximately 22% by day 4. The US group didn't show significant improvement compared to the Model group (*p* > 0.05), indicating that ultrasound alone has no therapeutic effect. In contrast, the HA-MN group exhibited a slight reduction in swelling by day 5, which may be attributed to the inherent anti-inflammatory properties of hyaluronic acid (Lee et al. 2021). Compared to the Cur‑aggregates@MN group, the Cur-PNPs@MN group exhibited markedly enhanced efficacy, underscoring the role of nanoparticle encapsulation in improving cellular uptake and retention at inflammatory sites and overcoming the limited bioavailability and poor solubility of unformulated curcumin. Both the Colchicine group and the SDT group (Cur-PNPs@MN + US) exhibited strong anti-inflammatory effects, with swelling rates nearing 9.1% ± 1.5% and 7.3% ± 1.2%, respectively, indicating that the sonodynamic therapy group not only matched but surpassed the efficacy of the conventional drug.

Representative photographs of rat ankles following treatment are presented in Figure 11C. Severe swelling persisted in the Model, US, HA-MN, and Cur‑aggregates@MN groups, while the Cur-PNPs@MN group showed moderate improvement. Notably, the SDT and Colchicine groups displayed ankle appearances comparable to the healthy Control group, further supporting the potent therapeutic effect of the combined nano-sonodynamic approach.

**Figure 11.**
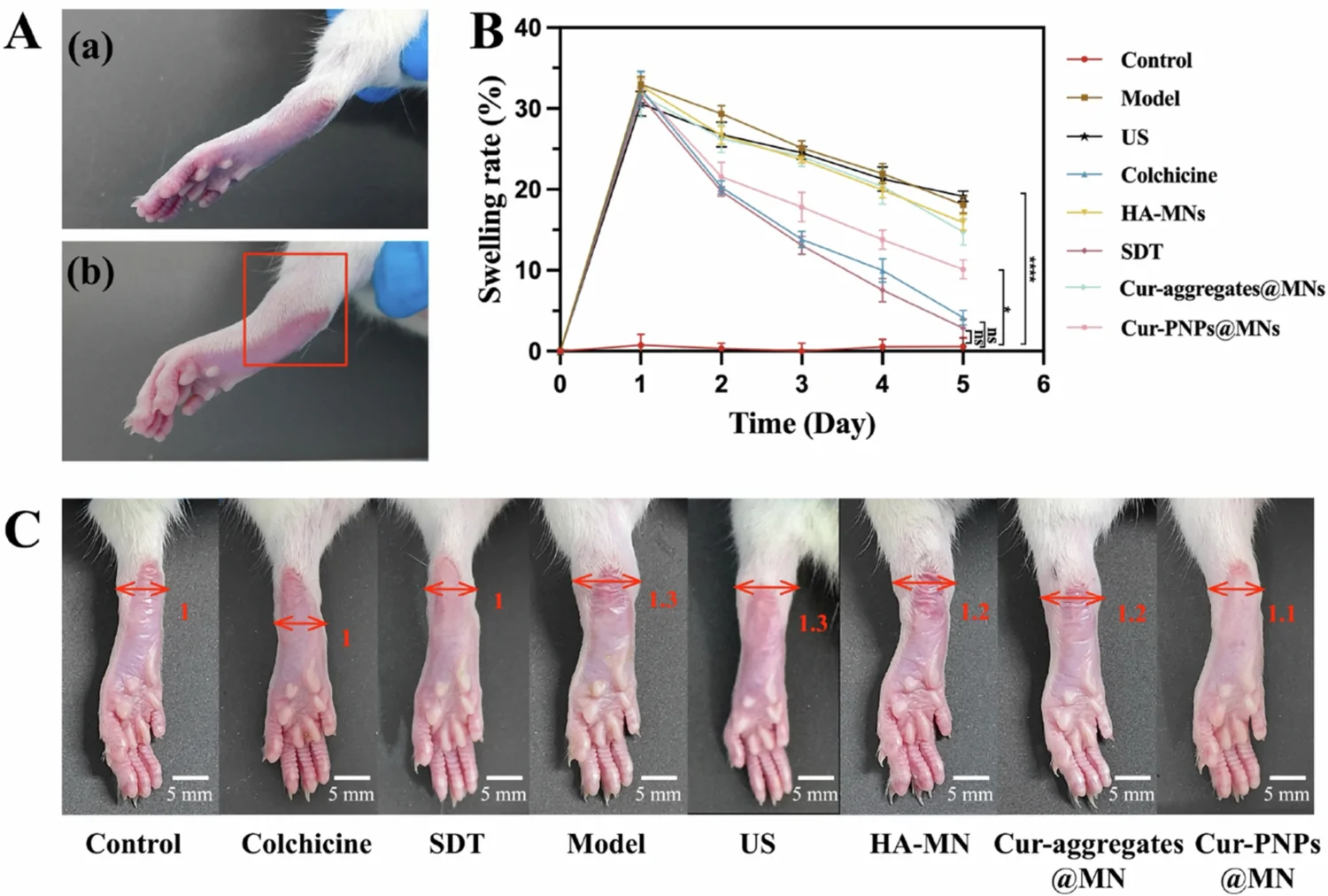
Changes in joint swelling of rats after modeling and treatment (*n* = 6 each group). (A). Effect of MSU on joint swelling (a: Injection of normal saline; b: Injection of MSU suspension). (B). Trends in joint swelling changes in rats of different groups. (C). Observation of joint swelling in rats of each group after the end of drug administration. Statistical analysis was performed using two-way ANOVA with Tukey's post-hoc test.

#### 3.11.2. Study of the improvement in inflammation

Massive neutrophil infiltration into the joint space is a hallmark pathological feature of gouty arthritis (Lei et al. 2022). To histologically evaluate the anti-inflammatory effects of the different treatments, ankle joint sections from each group were stained with hematoxylin and eosin (H&E). As shown in Figure 12A, the joint cavity in the Control group exhibited a smooth structure with no signs of inflammatory cell infiltration. In contrast, the Model group displayed substantial neutrophil infiltration and synovial hyperplasia characterized by thick, finger-like projections into the joint space. Both the US and HA-MN group showed a similar inflammatory profile to the Model group, indicating no therapeutic benefit from ultrasound alone and HA-MN alone. The Cur‑aggregates@MN groups still exhibited considerable inflammatory cell infiltration with no marked improvement in synovitis. The Cur-PNPs@MN group showed a partial reduction in cell infiltration, but the tidemark remained unclear. Notably, the SDT group (Cur-PNPs@MN + US) and the Colchicine group both displayed almost normal joint architecture with minimal inflammatory cell presence, demonstrating that sonodynamic therapy achieved anti-inflammatory efficacy comparable to that of the gold standard clinical reference drug.

From the perspective of inflammatory cytokine expression, the serum levels of IL-1β, IL-6, and TNF-*α* further confirmed the therapeutic outcomes (Figure 12B). Compared with the Control group, MSU stimulation significantly elevated serum concentrations of IL-1β (from 170.3 ± 14.5 to 67.3 ± 6.2 pg/mL), IL-6 (from 140.9 ± 12.6 pg/mL to 48.2 ± 4.5 pg/mL), and TNF-*α* (from 20.8 ± 2.1 pg/mL to 5.9 ± 0.6 pg/mL) in the Model group. The US group showed cytokine levels similar to those of the Model group, further supporting the limited effect of ultrasound alone. Minor reductions in inflammatory factors were observed in the HA-MN and Cur‑aggregates@MN groups, though these changes were not statistically significant. In contrast, the Cur-PNPs@MN group showed a more pronounced decrease in all three cytokines. Most importantly, the SDT group exhibited cytokine levels equivalent to those of the Control group, with no significant difference, and outperformed the colchicine group in suppressing TNF-*α*, IL-1β, and IL-6 expression.

Together, these findings indicate that SDT not only significantly alleviates neutrophil infiltration and synovial damage but also effectively downregulates MSU-induced pro-inflammatory cytokines, demonstrating superior anti-inflammatory activity relative to both conventional drug treatment and other experimental groups. These results highlight the promising potential of SDT as a targeted and efficient strategy for managing AGA.

**Figure 12.**
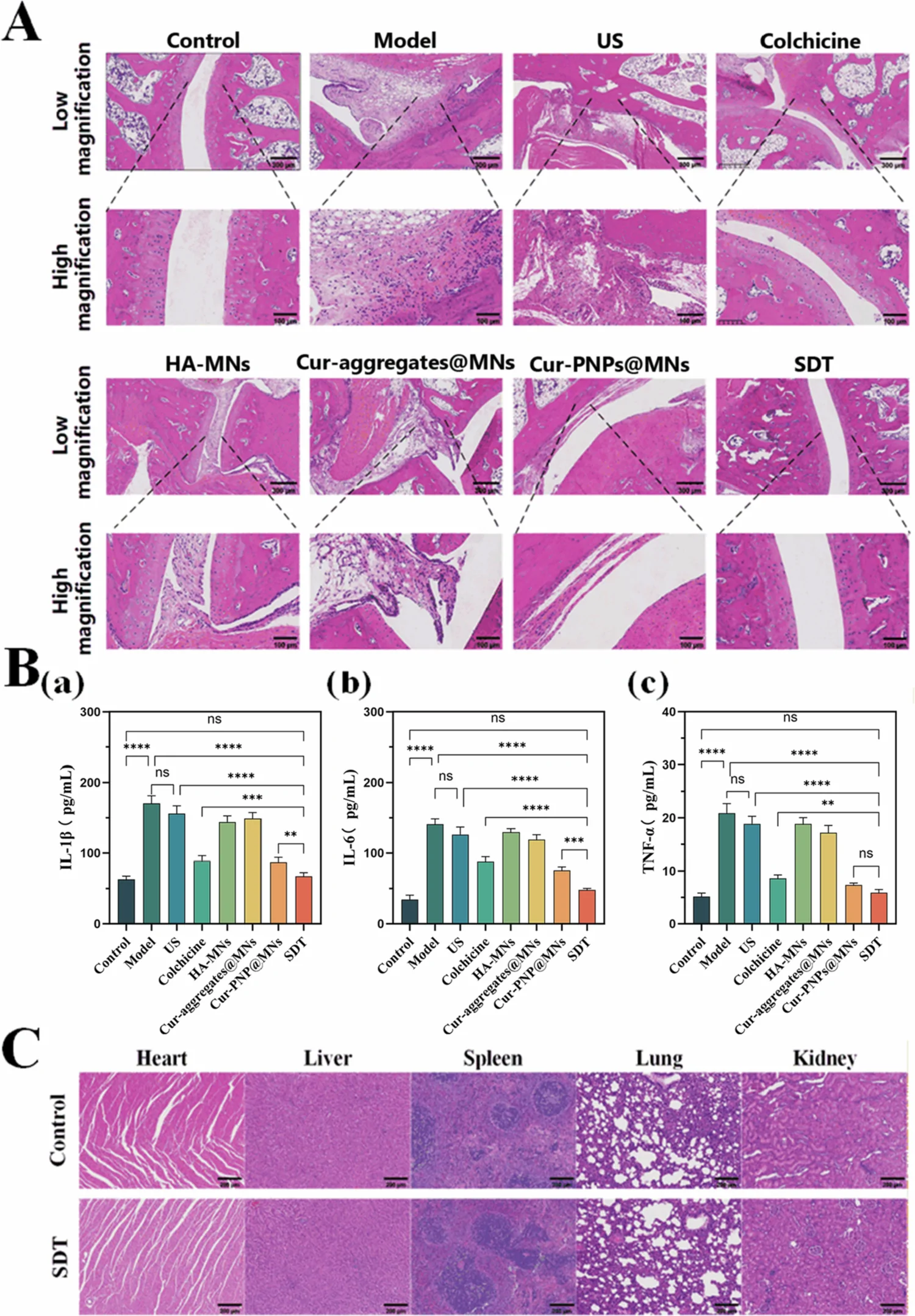
(A) H&E staining of ankle joints in rats of different groups (Scale bar: Low magnification: 300 μm; High magnification: 100 μm). (B) Levels of inflammatory cytokines IL-1β (a), IL-6 (b), and TNF-*α* (c) in rat serum after treatment. (C) H&E staining results of rat organs (heart, liver, spleen, lung, and kidney) in Control and SDT groups (Scale bar: 200 μm), (*n* = 6 each group). Statistical analysis was performed using two-way ANOVA with Tukey's post-hoc test.

#### 3.11.3. In vivo safety evaluation

Finally, major organs including heart, liver, spleen, lung, and kidney were collected from rats for H&E staining to evaluate the *in vivo* biocompatibility of the drug-loaded microneedle combined with ultrasound treatment. Consistent with previous *in vitro* biocompatibility assays, which showed that all components of the drug-loaded microneedles were non-toxic to rat fibroblasts, the results of organ H&E staining further supported this conclusion. As shown in Figure 12C, the tissue sections from both the normal group and the SDT group exhibited normal cellular morphology and no signs of pathological damage. These results confirm that the drug components in the microneedle patch are non-toxic to rats and demonstrate good biocompatibility *in vivo*.

## 4. Conclusion

In this study, we successfully developed a dissolvable microneedle-based transdermal system for synergistic sonodynamic and chemotherapy in the treatment of gouty arthritis. By encapsulating curcumin within acid-sensitive PCADK nanoparticles and incorporating them into microneedles, this platform enabled efficient, localized, and painless delivery of the hydrophobic sonosensitizer directly to the joint. The combination of nanocarrier-mediated solubilization and microneedle-facilitated transdermal release significantly enhanced the bioavailability and maximum deliverable dose of curcumin, while the acid-responsive properties of PCADK promoted lysosomal escape and nuclear translocation of the drug within macrophages. Under ultrasound activation, the elevated intracellular curcumin concentration led to robust, dose-dependent ROS generation, resulting in significantly improved sonodynamic efficacy. Both *in vitro* and *in vivo* evaluations demonstrated superior therapeutic outcomes: the system enhanced cellular uptake and subcellular targeting, and in a rat model of AGA, the microneedle-mediated SDT group outperformed conventional clinical treatment, showing marked reductions in joint inflammation and systemic cytokine levels. These findings underscore the potential of this integrated strategy—combining microneedle technology, nanotechnology-enhanced drug delivery, and ultrasound-triggered therapy—as a highly effective, targeted, and patient-compliant approach for the treatment of gouty arthritis.

## Supplementary Material

ARRIVE_Guidelines_Checklist.pdf ARRIVE_Guidelines_Checklist.pdf

## Data Availability

All data generated or analyzed during this study are included in this submitted article. The raw data will be made available on request.
